# Distinct Roles of RNA Helicases MVH and TDRD9 in PIWI Slicing-Triggered Mammalian piRNA Biogenesis and Function

**DOI:** 10.1016/j.devcel.2017.05.021

**Published:** 2017-06-19

**Authors:** Joanna M. Wenda, David Homolka, Zhaolin Yang, Pietro Spinelli, Ravi Sachidanandam, Radha Raman Pandey, Ramesh S. Pillai

**Affiliations:** 1 Department of Molecular Biology, University of Geneva, 30 Quai Ernest-Ansermet, 1211 Geneva, Switzerland; 2 European Molecular Biology Laboratory, Grenoble Outstation, 71 Avenue des Martyrs, 38042 Grenoble, France; 3 Department of Oncological Sciences, Icahn School of Medicine at Sinai, One Gustave L. Levy Place, New York, NY 10029, USA

**Keywords:** Mvh, Tdrd9, Vasa, Spn-E, Piwi, spermatogenesis, Ddx4, Miwi, Mili, piRNAs

## Abstract

Small RNAs called PIWI-interacting RNAs (piRNAs) act as an immune system to suppress transposable elements in the animal gonads. A poorly understood adaptive pathway links cytoplasmic slicing of target RNA by the PIWI protein MILI to loading of target-derived piRNAs into nuclear MIWI2. Here we demonstrate that MILI slicing generates a 16-nt by-product that is discarded and a pre-piRNA intermediate that is used for phased piRNA production. The ATPase activity of Mouse Vasa Homolog (MVH) is essential for processing the intermediate into piRNAs, ensuring transposon silencing and male fertility. The ATPase activity controls dissociation of an MVH complex containing PIWI proteins, piRNAs, and slicer products, allowing safe handover of the intermediate. In contrast, ATPase activity of TDRD9 is dispensable for piRNA biogenesis but is essential for transposon silencing and male fertility. Our work implicates distinct RNA helicases in specific steps along the nuclear piRNA pathway.

## Highlights

• MILI slicing of an RNA creates a pre-piRNA intermediate and a 16-nt by-product
• ATPase activity of helicase MVH is required for the pre-piRNA to mature as a piRNA
• ATPase mutant MVH traps biogenesis factors, piRNAs, and slicer products
• Helicase TDRD9 is essential for transposon silencing, but not piRNA biogenesis

## Graphical Abstract

**Figure:**
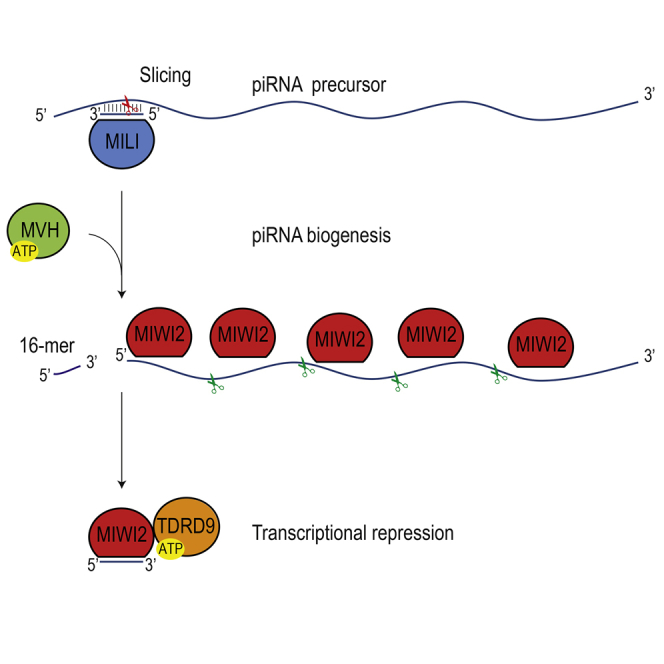

## Teaser

PIWI-interacting RNAs (piRNAs) are gonad-specific small RNAs targeting transposon and cellular mRNAs and are essential to male mouse fertility. Wenda et al. uncover sequential roles for RNA helicases in piRNA biogenesis and function: MVH is essential for maturation of PIWI slicer products to piRNAs, whereas TDRD9 is essential for transposon silencing.

## Introduction

PIWI-interacting RNAs (piRNAs) are animal gonad-specific 24- to 30-nt small RNAs that associate with Argonaute proteins of the PIWI clade (Luteijn and Ketting, 2013). The basic functional unit consists of a single-stranded piRNA molecule complexed with a PIWI protein, where the small RNA acts as a guide for the protein by selecting nucleic acid targets via sequence complementarity (Matsumoto et al., 2016). Cytosolic mouse PIWI proteins MIWI and MILI are piRNA-guided endoribonucleases or slicers that cleave target transcripts for silencing (De Fazio et al., 2011, Reuter et al., 2011), while nuclear PIWI protein MIWI2 is proposed to recruit the histone or DNA methylation machinery to target genomic loci for transcriptional repression (Aravin et al., 2008, Kojima-Kita et al., 2016, Kuramochi-Miyagawa et al., 2008).

Mobile genetic elements or transposons constitute the universal target of the piRNA pathway (Ghildiyal and Zamore, 2009). In the mouse embryonic male germline, MILI and MIWI2 are loaded with repetitive transposon-silencing piRNAs (Aravin et al., 2007, Aravin et al., 2008). However, transposons are not the only targets for piRNAs in the adult germline, where MILI and MIWI bind an abundant set of non-repetitive unique sequences that are collectively called pachytene piRNAs (Aravin et al., 2006, Girard et al., 2006, Li et al., 2013, Vourekas et al., 2015). These are shown to promote selective removal of transposon or cellular mRNAs by guiding PIWI slicing or facilitating the recruitment of the mRNA deadenylation machinery, or by regulating translation (Castaneda et al., 2014, Goh et al., 2015, Gou et al., 2014, Reuter et al., 2011, Zhang et al., 2015). Thus, both transposon and cellular mRNAs are targets of the mouse piRNA pathway, and their regulation is essential for spermatogenesis and fertility in male mice (Pillai and Chuma, 2012).

Large genomic regions called piRNA clusters are sources for a majority of the piRNAs (Aravin et al., 2006, Aravin et al., 2008, Girard et al., 2006). In addition to clusters, individual transposons and a selected set of cellular transcripts are also used as substrates for piRNA biogenesis. After transcription by RNA polymerase II, these precursors are exported to the cytoplasm where they meet up with the biogenesis machinery resident in perinuclear cytoplasmic granules called nuage (Aravin et al., 2009, Li et al., 2013). The processing machinery cleaves the single-stranded precursors into thousands of non-overlapping/phased fragments, each of which is loaded into a PIWI protein where it matures as a piRNA after 3′ end processing (Han et al., 2015, Homolka et al., 2015, Izumi et al., 2016, Mohn et al., 2015, Saxe et al., 2013). The resulting piRNAs have a strong preference for having a 5′ uridine (U1 bias).

How the precursors are specifically selected for processing is not completely understood. Genetic studies point to two distinct pathways that are in operation in the mouse male germline. A default pathway called primary processing recruits precursors, by an unknown mechanism, into the biogenesis machinery for generation of majority of the piRNAs that associate with MILI and MIWI (Li et al., 2013, Vourekas et al., 2012, Zheng and Wang, 2012). A second pathway uses MILI slicing to identify a target RNA as a substrate for piRNA generation, leading to extensive conversion of one of the cleavage fragments into non-overlapping/phased piRNAs (Han et al., 2015, Mohn et al., 2015, Saxe et al., 2013, Yang et al., 2016). Although MILI slicing loads both MILI and MIWI2 with the target-derived piRNAs (Yang et al., 2016), MIWI2 is totally dependent on this pathway to acquire its small RNA guides (Aravin et al., 2008, De Fazio et al., 2011, Kuramochi-Miyagawa et al., 2008). The use of PIWI slicing as an initiator of piRNA biogenesis on a transcript is mechanistically challenging, as slicing is also used for target destruction. How the slicer cleavage fragment is safely handed over to the biogenesis machinery in the mouse male germline is not currently understood. Attesting to the complexity of the pathway, a number of piRNA biogenesis factors are exclusively required for slicer-triggered piRNA biogenesis. These include the RNA helicase Mouse Vasa Homolog (MVH) (Kuramochi-Miyagawa et al., 2010), Tudor domain proteins TDRD1 (Reuter et al., 2009) and TDRD12 (Pandey et al., 2013), the co-chaperone FKBP6 (Xiol et al., 2012), and the TDRD12 partner EXD1 (Yang et al., 2016).

In this study, we demonstrate that ATPase activity of MVH (Tanaka et al., 2000) is essential for MILI slicing-triggered piRNA biogenesis. Using an artificial piRNA precursor, we find that ATPase activity of MVH enables utilization of one of the slicer cleavage fragments for phased piRNA generation. Loss of this activity leads to accumulation of the piRNA intermediate, and affects transposon silencing and fertility in male mice. Examination of a second RNA helicase, TDRD9 (Shoji et al., 2009), shows that its ATPase activity is dispensable for piRNA biogenesis but is essential for transposon silencing and fertility. Taken together, our work reveals how distinct RNA helicases use their ATPase activities to participate in specific steps along the mammalian nuclear piRNA pathway.

## Results

### MILI Slicing Generates a Pre-piRNA Intermediate and a 16-nt By-product

**Figure 1:**
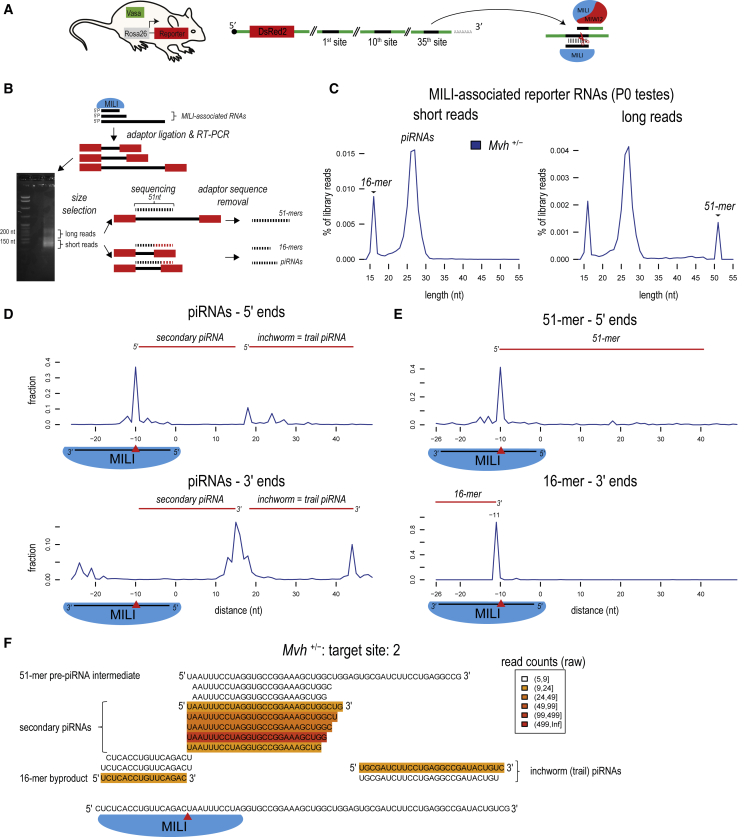
MILI Slicing Generates a Pre-piRNA Intermediate and a 16-nt By-product (A) Design of a mouse reporter expressing an artificial piRNA precursor consisting of the DsRed2 coding sequence with a 3′ UTR carrying 35 perfectly complementary binding sites for MILI-bound piRNAs that are expressed in the embryonic mouse male germline (see STAR Methods). This reporter was crossed into the various *Mvh* genetic backgrounds, and MILI slicing-triggered piRNA biogenesis from the reporter was examined. See also Figure S1. (B) MILI-bound RNAs were isolated from testes of neonatal (P0) *Mvh*^*+*/*−*^ animals and cDNA libraries prepared by ligating adapters that depend on presence of a 5′ phosphate on the RNAs. The library was separated into two size ranges: short reads (with insert of ∼10–30 nt) and long reads (of ∼20–100 nt), and sequenced independently for 51 cycles. After the removal of 3′ adaptor sequences, we obtained 51-nt long reads representing the 5′ end of long RNAs and short reads corresponding to complete sequences of small RNAs. (C) Read-length distribution of reads derived from the reporter is shown. Note that 51 nt corresponds to the maximum sequencing length. (D) Mapping of the 5′ and 3′ ends of reporter-derived RNAs relative to the 5′ end (nucleotide position −1) of the targeting MILI piRNA. Position −10 refers to the MILI cleavage site (red arrowhead), which is guided by the targeting piRNA. The counts were aggregated for the 35 individual MILI-targeted sites. The secondary piRNAs have their 5′ end created by the MILI slicing at position −10, while the inchworm (or trail) piRNAs start downstream of the 3′ ends of secondary piRNAs. See also Table S1. (E) The 5′ end of 51-mers map to the MILI cleavage sites (red arrowhead) and therefore represent the pre-piRNA intermediates. The 16-mers represent the slicer by-product, as it has its 3′ end created by MILI cleavage, but is not used for piRNA generation. (F) Sequence-level details of individual MILI-associated RNAs produced in the vicinity of the second target site on the reporter are shown. The red arrowhead indicates the MILI cleavage site.

MILI slicing-triggered biogenesis pathway is most active in the embryonic/perinatal male germline in mice. Endogenous piRNAs guiding MILI, and their targets in this environment are all highly repetitive, complicating their study by sequence analysis. To overcome this, we previously created a knockin mouse line (*Rosa26-pi*^*KI*^) expressing an artificial piRNA precursor which is targeted by endogenous MILI (Yang et al., 2016). In brief, it consists of a dsRed reporter with a 3′ UTR based on non-coding LacZ sequence, where we inserted perfectly complementary binding sites for 35 independent MILI-bound piRNAs that are abundant in the mouse embryonic germline (Figure 1A) (see STAR Methods). MILI slicing of the precursor triggered production of a series of piRNAs from the reporter, which were loaded into both MIWI2 and MILI (Yang et al., 2016). This confirmed the reporter as a useful tool for examining the consequences of MILI slicing.

To directly examine the cleavage fragments generated by MILI slicing, we isolated RNAs present in MILI complexes from newborn pups (post-natal day 0 [P0]) (*Mvh*^*+*/*−*^; *Rosa26-pi*^*KI*^ genotype) (STAR Methods). After reverse transcription, the cDNAs were resolved in a gel and fragments roughly corresponding to RNAs of 10–30 nt (short reads) and 30–50 nt (long reads) were sequenced separately (Figures 1B and S1). The reads were sequenced to a maximum length of 51 nt. After mapping to the reporter sequence, only perfectly matching reads were considered for further analysis (Table S1). Read-length distribution shows that both short- and long-read libraries contain a prominent peak at 26 nt corresponding to the length of MILI piRNAs (Figure 1C), and a second peak at 16 nt of unknown origin. Additionally the long-read library had a third peak at 51 nt, which corresponds to the sequenced 5′ end portion of longer RNAs.

PIWI slicing cleaves a target RNA at a site 10 nt downstream of the 5′ end of the guiding piRNA (Reuter et al., 2011). This generates two fragments, one with its 3′ end and another with its 5′ end, at the site of cleavage. The fragment with the 5′ end is shown to mature as a new secondary piRNA (Aravin et al., 2008, Brennecke et al., 2007, Gunawardane et al., 2007). To precisely identify the origin of the various reporter-derived sequences, we mapped the reads to the reporter sequence and calculated their 5′ and 3′ end distances with respect to the 5′ end of the targeting piRNA. This indicates that majority of the reporter-derived piRNAs (24–30 nt) have their 5′ ends (at −10 position) generated by MILI slicing, and therefore are termed secondary piRNAs (Figure 1D). We also detect a second set of 5′ ends (peaks at positions 18–26), which are not generated by MILI slicing. These correspond to the inchworm or trail piRNAs that arise immediately downstream of a secondary piRNA (Homolka et al., 2015, Mohn et al., 2015, Yang et al., 2016) (Figure 1D). The 3′ ends of both piRNAs can be identified at distances corresponding to the size of mature MILI-bound piRNAs (∼26 nt) (Figure 1D). It is expected that MILI slicing initiates the generation of a series of non-overlapping/phased piRNAs in the 5′ → 3′ direction that are loaded into MILI and MIWI2, as previously noted (Yang et al., 2016).

Next, we examined the novel fragments that were not observed previously, perhaps due to differences in the library preparation protocols (Figure 1B; see STAR Methods). The 51-mer sequences have their 5′ ends at the −10 position, indicating their generation by MILI slicing. Thus, these are pre-piRNA intermediates that have the same 5′ ends as the mature secondary piRNAs, but have extended 3′ ends (Figure 1E). It is likely that after MILI slicing, these are handed over to the piRNA biogenesis machinery that produces a series of phased piRNAs. Mapping of the 16-mers to the reporter reveals that they arise from the upstream MILI cleavage fragment that is normally not used for piRNA generation (Figure 1E). This identifies the 16-mers as by-products of piRNA biogenesis. They are perfectly complementary to the trigger piRNAs and have a precise 3′ end that lies at the site of MILI slicing (peak at position −10). Being protected within the MILI complex, the 5′ ends of the 16-mers are compatible with their generation by an unknown nuclease that shortens the cleavage fragment by generating a footprint of MILI. Thus, MILI endonuclease action on a target RNA generates two fragments with distinct fates (Figure 1F): one becomes a pre-piRNA intermediate that is destined for phased piRNA generation while the other is processed into a 16-nt by-product that is likely released from the complex and degraded.

### ATPase Activity of MVH Is Essential for Spermatogenesis and Transposon Control in *Mvh*^*−*/*KI*^ Mice

**Figure 2:**
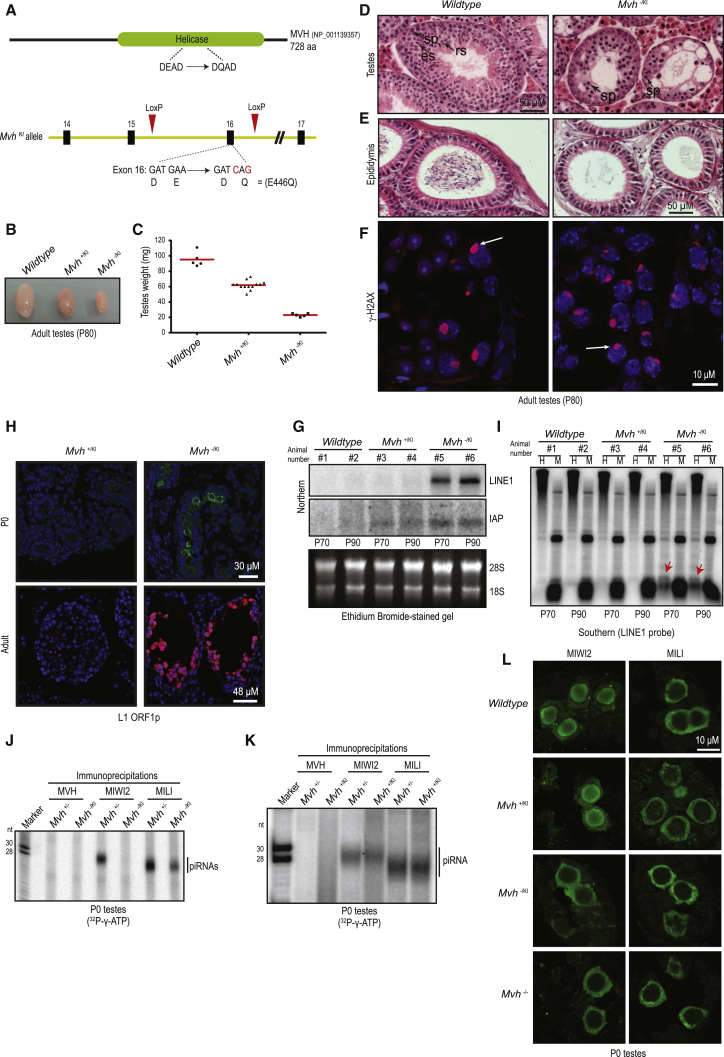
Catalytic Activity of MVH Is Essential for Transposon Silencing and Biogenesis of MIWI2 piRNAs (A) Creation of the catalytic-dead *Mvh* mouse carrying a point mutation E446Q in the ATPase motif (DEAD → DQAD). See also Figure S2. (B) Representative testes from adult animals (P80; 80 days old) of indicated *Mvh* genotypes. (C) Testes weight in different genotypes. (D and E) H&E staining of adult mouse testes showing arrested germ cell development in the *Mvh*^*−*/*KI*^ mutant (D), and (E) presence of sperm in the lumen of the wild-type epididymis, but not from that of the mutant. sp, spermatocytes; rs, round spermatids; es, elongated spermatids. Scale bars, 50 μm. (F) Staining for γ-H2AX in adult testes sections. Arrows point to the XY body. Scale bar, 10 μm. (G) Northern analysis for indicated transposon transcripts in total testicular RNA. The donor animals are numbered and their ages indicated. Total testicular DNA from the same animals were used for Southern blotting in (I). (H) Staining for L1ORF1p in mouse testes from animals of indicated ages. Scale bars, 38 μm (upper) and 48 μm (lower). (I) Methylation-sensitive Southern blotting examining L1 genomic loci. The donor animals are the same as those used for northern analysis (indicated by animal numbers). The red arrows point to the cleavage fragment seen under conditions of reduced DNA methylation, and only in the *Mvh*^*−*/*KI*^ mutant. H, HpaII-digested DNA; M, MspI-digested DNA. (J and K) Immunoprecipitation of PIWI proteins from neonatal (P0) testes and 5′ end labeling of associated piRNAs. RNA size markers are 5′ end labeled (length in nucleotides). (L) Immunofluorescence detection of indicated proteins in neonatal testes Scale bar, 10 μm.

Given the overlapping roles of MILI slicing in target degradation and piRNA biogenesis, there must be mechanisms to protect the pre-piRNA intermediate from degradation and safely transfer it to the biogenesis machinery. RNA helicases are ATP-driven machines that mediate dynamic interactions with their protein and RNA partners (Linder and Jankowsky, 2011), and are potential mediators of such transactions. The conserved RNA helicase MVH is required for biogenesis of MIWI2 piRNAs (Kuramochi-Miyagawa et al., 2010, Tanaka et al., 2000), and we wished to examine its molecular role in the pathway. We created a knockin mouse mutant (referred to as *Mvh*^*KI*^) that carries a point mutation E446Q in its ATPase motif (DEAD → DQAD) (STAR Methods; Figures 2A and S2). A similar mutation in the *Bombyx mori* (silkworm) Vasa disrupts its activity (Xiol et al., 2014). The design of our mouse mutant also allowed us to create mice carrying the knockout allele (referred to as *Mvh*^*−*^).

As described previously for an independent *Mvh* null mutant (Tanaka et al., 2000), our homozygous knockout mutant (*Mvh*^*−*/*−*^) males are infertile. The heterozygous *Mvh*^*+*/*KI*^ mutant, where both the wild-type and catalytic-dead mutant MVH^E446Q^ proteins are co-expressed, also displays male-specific infertility. This indicates a dominant-negative effect of the mutation. To obtain a clean system to study the impact of the catalytic-dead mutation in *Mvh*, we created the hemizygous *Mvh*^*−*/*KI*^ mutant by intercrosses with mice carrying the *Mvh*^*−*^ knockout allele. The *Mvh*^*−*/*KI*^ males are also infertile and reveal highly atrophied testes (Figures 2B and 2C). This phenotype is very similar to the homozygous null mutant (Tanaka et al., 2000). Females of all *Mvh* genotypes are fertile. Examination of testes sections from adult (60-day-old mice; P60) *Mvh*^*−*/*KI*^ animals revealed an arrested spermatogenesis characterized by lack of late-stage germ cells, namely round spermatids (Figure 2D). Consequently, mature sperm is not detected in the epididymis of the *Mvh*^*−*/*KI*^ mutants (Figure 2E). In meiotic pachytene spermatocytes, the unsynapsed sex chromosomes form a structure called the sex body, which is decorated by the phosphorylated form of the histone variant H2AX (γ-H2AX). Staining of testes sections reveals the presence of the sex body in both the wild-type and *Mvh*^*−*/*KI*^ mutant, indicating proper progression of spermatogenesis until pachynema (Figure 2F). The arrested germ cells are then eliminated by apoptosis (Figure S4B), resulting in narrow, empty seminiferous tubules in the *Mvh*^*−*/*KI*^ testes (Figure 2D).

The piRNA pathway controls two retrotransposons in the mouse genome: long terminal repeat (LTR) type IAP and the non-LTR LINE1 (L1). Given the spermatogenic defect, we examined expression of these elements in the *Mvh* knockin mutant testes. Northern analysis with testicular total RNA indicates strong expression of L1 transcripts in duplicate biological replicates of the *Mvh*^*−*/*KI*^ mutant (Figure 2G). The levels of IAP retrotransposons are only mildly increased. These L1 transcripts are from functional copies of the transposon, as a translation product (L1ORF1p) can be detected by immunofluorescence in germ cells of both P0 and adult *Mvh*^*−*/*KI*^ mutants (Figure 2H). Cytosine DNA methylation is shown to suppress transposable elements in the mammalian genome (Nagamori et al., 2015). Examination of DNA methylation on L1 genomic loci by methylation-sensitive Southern blotting indicates reduced methylation of L1 regions in the *Mvh*^*−*/*KI*^ mutants (Figure 2I), explaining their activated status. We note that the activation of transposons is similar to that observed in the null mutant (Kuramochi-Miyagawa et al., 2010). Surprisingly, although infertile, we did not observe any derepression of transposons in the heterozygous *Mvh*^*+*/*KI*^ mutants, and consistently no changes were noted in L1 DNA methylation levels (Figures 2G–2I). Taken together, we show that ATPase activity of MVH is essential for fertility and transposon control in *Mvh*^*−*/*KI*^ male mice.

### Transposon Activation in Catalytic-Dead *Mvh*^*−*/*KI*^ Mutants Is Due to Loss of MIWI2 piRNAs

Activation of transposons and the loss of DNA methylation on their genomic loci is indicative of a failure of the piRNA pathway in the *Mvh*^*−*/*KI*^ mutant. Transposon silencing by piRNAs is orchestrated in the embryonic/perinatal male germline by a tight coordination between cytosolic MILI and nuclear MIWI2. To ascertain the integrity of the piRNA pathway, we isolated PIWI protein complexes from P0 testes, and examined the presence of small RNAs by 5′ end labeling. While the control *Mvh*^*+*/*−*^ animals revealed normal association of MILI and MIWI2 with ∼26-nt and ∼28-nt piRNAs, respectively, only MILI was loaded with piRNAs in the *Mvh*^*−*/*KI*^ mutant (Figure 2J). This lack of MIWI2 piRNAs is similar to that reported for the *Mvh* null mutant (Kuramochi-Miyagawa et al., 2010). Consistent with the lack of transposon derepression, the *Mvh*^*+*/*KI*^ mutant did not reveal any deficiencies in piRNA association (Figure 2K). We report that deep-sequencing analysis indicates the presence of an unchanged population of MIWI2 piRNAs in the *Mvh*^*+*/*−*^ and *Mvh*^*+*/*KI*^ animals (Figures S3A–S3C). As nuclear localization of MIWI2 is licensed by its binding to piRNAs (Aravin et al., 2008), unloaded MIWI2 in P0 *Mvh*^*−*/*KI*^ mutant testes is retained in the cytoplasm (Figure 2L). A similar situation is observed in the *Mvh* null mutant we generated (Figure 2L). The piRNA-loaded MIWI2 is nuclear in wild-type and *Mvh*^*+*/*KI*^ mutant animals, while cytosolic localization of MILI remains unchanged in all genotypes (Figure 2L). Thus, transposon activation in the MVH catalytic-dead *Mvh*^*−*/*KI*^ mutant mice is a result of impaired biogenesis of MIWI2 piRNAs.

### Loss of Repeat piRNAs in the *Mvh*^*−*/*KI*^ Mutant

**Figure 3:**
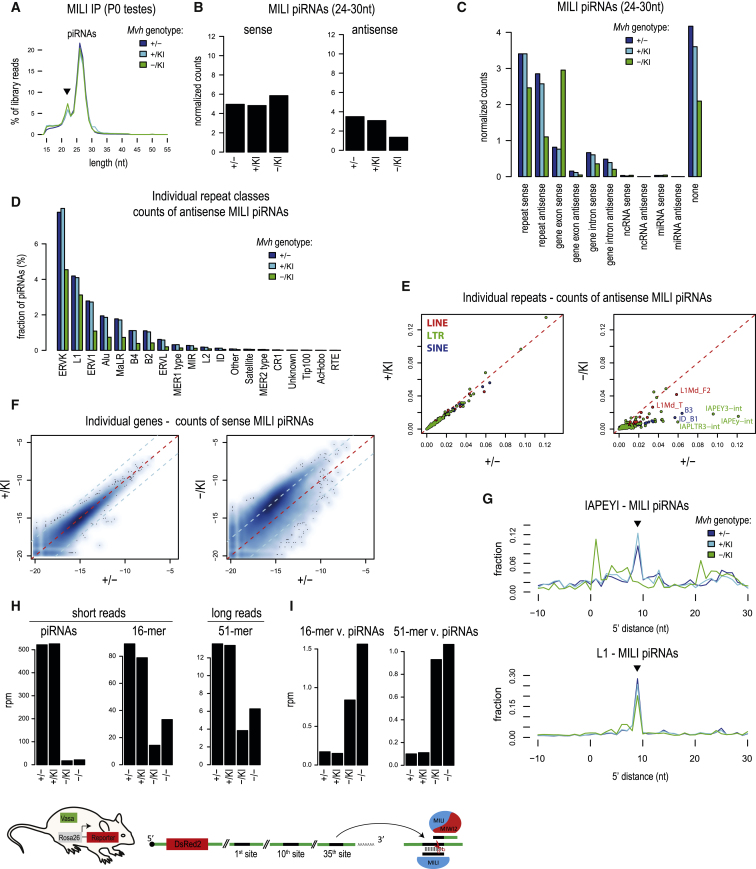
Inability to Convert Pre-piRNA Intermediates Generated by MILI Slicing into piRNAs in the *Mvh*^*−*/*KI*^ Mutant (A) Length distributions of MILI-bound RNAs from neonatal (P0) testes. Majority of the reads refer to piRNAs (26- to 27-nt peak). A contaminating population of miRNAs is marked by arrowhead (22-nt peak). (B) MILI-associated piRNAs of 24- to 30-nt size range were divided into “sense” (originating from annotated transcripts) and “antisense” (targeting the transcripts). Their counts were normalized to contaminating miRNAs and compared. The *Mvh*^−/KI^ mutant shows an overall decrease of “antisense” piRNAs. See also Figure S3. (C) Most of the piRNA classes are depleted in *Mvh*^−/KI^, with the antisense piRNAs being preferentially affected. The depletion is accompanied by an increased proportion of piRNAs originating from genic transcripts (gene exon sense). (D) Decrease in antisense repeat piRNAs in the *Mvh*^−/KI^ mutant for most of the top 20 repeat classes. (E) Normalized counts of antisense piRNAs were compared for individual repeats. Strong reduction of antisense piRNAs is observed for many of the repeats in the *Mvh*^−/KI^ mutant. The levels of piRNAs targeting the IAPEY repeats are especially affected. (F) The normalized counts of sense-oriented genic piRNAs are compared for individual genes. The color density representation of the scatterplot shows the overall increase (∼5-fold) of genic piRNAs in *Mvh*^−/KI^. Red line, no change; blue line, 5-fold difference. (G) Plots show the preferred 5′ end distances between the targeting and produced piRNAs for IAPEY and L1 transposons. The ping-pong signature (arrowhead) is decreased for the mapped L1 piRNAs, and absent for IAPEYI piRNAs, in the *Mvh*^−/KI^ mutant. (H) The amount of MILI-associated RNAs produced from the artificial piRNA precursor (*Rosa26-pi*) was compared between the wild-type and the various *Mvh* mutant genotypes. The reporter-derived piRNAs are drastically depleted in the *Mvh*^−/KI^ and *Mvh*^−/−^ mutants. The 16-mers and 51-mers also display a reduction in the mutants, although not comparable with the decrease of piRNAs. (I) The relative ratio of 16-mers and 51-mers (compared with piRNA levels within the same library) is increased in the *Mvh*^−/KI^ and *Mvh*^−/−^ mutants. See also Figure S1.

The piRNAs generated by MILI slicing are loaded into both MIWI2 and MILI (Yang et al., 2016). Given the loss of all MIWI2 piRNAs in the *Mvh*^*−*/*KI*^ mutant, we examined the MILI-bound population. MILI complexes were isolated from P0 testes of the *Mvh*^*−*/*KI*^ mutant and deep-sequencing libraries were prepared. For comparison, we prepared similar libraries from the *Mvh*^*+*/*−*^ and *Mvh*^*+*/*KI*^ animals. Analysis of read-length distribution in the libraries identifies the expected peak of ∼26-nt sequences, together with a contaminating peak of ∼22-nt microRNAs (miRNAs) (Figure 3A). The piRNA-sized reads (24–30 nt in length) were mapped to the mouse genome and annotations extracted. When examined across all annotation classes and normalized to the levels of miRNAs within each library, the overall levels of sense-oriented reads were increased, while sequences with an antisense orientation decreased in the *Mvh*^*−*/*KI*^ mutant (Figure 3B). When specific annotation classes were examined, we observed a sharp increase in gene exonic sense reads and a concomitant decrease in other classes (Figure 3C). Most of the repeat classes show a decrease in piRNA levels (Figures 3D and S3D). Examination at the level of individual repeats reveals that L1Md_F2, L1Md_T, IAPEY3-int, IAPEy-int, and IAPLTR3-int are the ones that suffer decreases in antisense piRNAs within MILI in the *Mvh*^*−*/*KI*^ mutant (Figures 3E, S3E, and S3F), which is consistent with the demonstrated derepression of these transposable elements in the mutant (Figures 2G–2I). In contrast, levels of repeat piRNAs in the *Mvh*^*+*/*KI*^ mutant are unchanged when compared with the control *Mvh*^*+*/*−*^ animals (Figure 3E). Interestingly, we noticed an increased (∼5-fold) occupancy of reads from cellular mRNAs in MILI ribonucleoprotein particles in the *Mvh*^*−*/*KI*^ mutant (Figure 3F). Given that MILI is the cytosolic endonuclease that mediates post-transcriptional silencing, together with the loss of MIWI2 piRNAs these changes contribute to reduced repression of transposons in the *Mvh*^*−*/*KI*^ mutant.

MILI slicing contributes to secondary piRNA biogenesis, and this can be monitored within piRNA populations by the presence of a 10-nt overlap (ping-pong signal) between 5′ ends of piRNAs. Indeed, when reads are aligned over the IAPEYI consensus sequence, a 10-nt overlap signal (corresponding to 9-nt 5′ end distance) is present in MILI-bound piRNAs from the control and *Mvh*^*+*/*KI*^ animals, but absent in the *Mvh*^*−*/*KI*^ mutant (Figure 3G). A decrease, albeit much smaller, is also observed when examined over the L1 consensus (Figure 3G). These results indicate that MILI slicing-triggered biogenesis of repeat piRNAs is affected in the *Mvh*^*−*/*KI*^ mutant.

### MILI Can Slice Targets in the *Mvh*^*−*/*KI*^ Mutant, but These Fail to Mature into piRNAs

To unambiguously examine the role of MVH in piRNA biogenesis driven by MILI slicing, we brought the artificial precursor (*Rosa26-pi*) into the different *Mvh* mutant genetic backgrounds (*Mvh*^*+*/*KI*^, *Mvh*^*−*/*−*^, and *Mvh*^*−*/*KI*^). Examination of reporter-derived sequences in MILI complexes (from P0 testes) reveals the presence of piRNAs, 16-mer by-products, and 51-mer pre-piRNA intermediates (Figure S1). These all bear signatures consistent with their origin via MILI slicing. When compared with the control lines (*Mvh*^*+*/*−*^ and *Mvh*^*+*/*KI*^), piRNA read counts were greatly diminished in the *Mvh*^*−*/*−*^ and *Mvh*^*−*/*KI*^ mutants, as expected (Figure 3H). Importantly, although the overall abundance of 16- and 51-mers was also affected in the *Mvh*^*−*/*−*^ and *Mvh*^*−*/*KI*^ mutants (Figure 3H), their relative abundance with respect to mature piRNAs was strongly elevated (Figure 3I). This indicates that MILI is able to identify and engage targets for slicing in the *Mvh*^*−*/*−*^ and *Mvh*^*−*/*KI*^ mutants, but in the absence of MVH or its catalytic activity this does not lead to productive conversion of the pre-piRNA intermediate fragment into piRNAs.

### Male Infertility in the Dominant-Negative *Mvh*^*+*/*KI*^ Mutant Is Independent of Transposon Activation

The aforementioned experiments demonstrate a role of the catalytic activity of MVH in facilitating MILI slicing-triggered piRNA biogenesis. They also highlight the consequences of not having functional MVH in the *Mvh*^*−*/*KI*^ mutant: transposon derepression and male-specific infertility. However, these observations do not explain why the dominant-negative heterozygous *Mvh*^*+*/*KI*^ mutant males are infertile, as they do not exhibit transposon derepression and have normal biogenesis of embryonic piRNAs.

**Figure 4:**
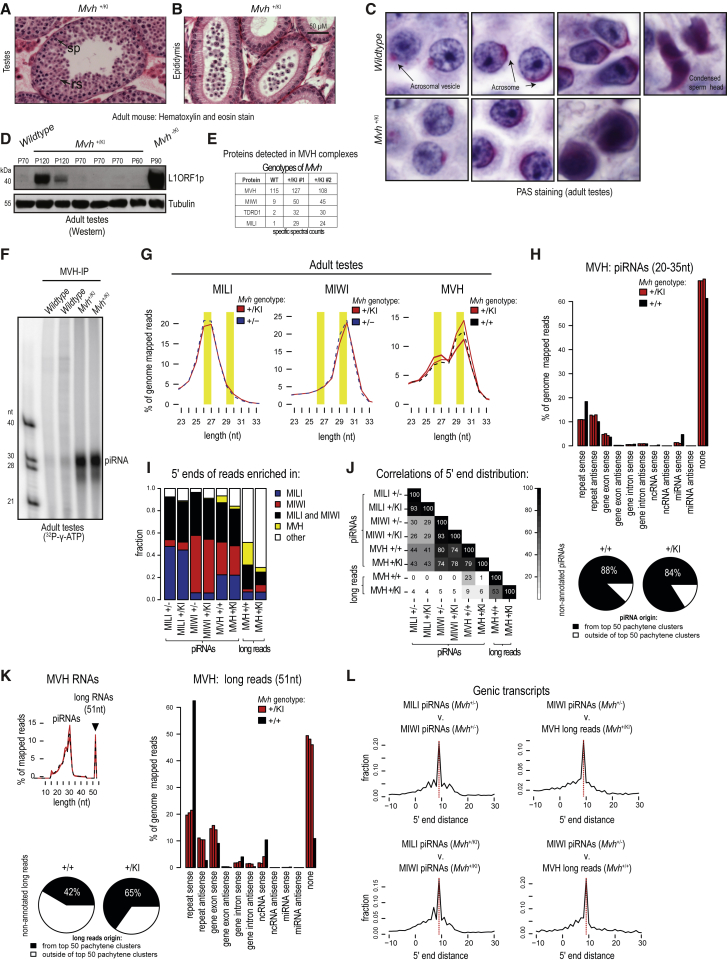
Catalytic-Dead MVH Dominant-Negative Mutant Traps Pachytene piRNAs, Biogenesis Factors, and Slicer Products (A) Histology of adult mouse testes showing arrested spermatogenesis in the *Mvh*^*+*/*KI*^ mutant, indicating that the catalytic-dead MVH protein acts as a dominant negative. Germ cells are arrested at round spermatids. sp, spermatocytes; rs, round spermatids. See also Figure S4. (B) Epididymis showing degenerated cells, but no mature sperm. Scale bar, 50 μm (applies also to A). (C) Periodic acid-Schiff (PAS) staining to detect developing acrosome in round spermatids in wild-type and the *Mvh*^+/KI^ mutant. Scale: the width of each image is 25 μm. (D) Western analysis of total testicular lysates to monitor expression of L1ORF1p. Tubulin is used as loading control. Age of donor animals indicated (P70; 70 days old). (E) Identification by mass spectrometry of protein components in isolated MVH complexes from adult testes of indicated genotypes. The specific spectral counts for the top four identified proteins are shown. WT, wild-type. (F) Immunoprecipitation of MVH from adult wild-type or *Mvh*^*+*/*KI*^ mutant testes, and 5′ end labeling of associated RNAs. Labeled single-stranded RNA markers are indicated (length in nucleotides). See also Figure S4E. (G) Length (in nucleotides) distributions of piRNAs associating with MILI, MIWI, and MVH in adult testes of indicated *Mvh* genotypes. MVH associates with RNAs with lengths typical (shaded in yellow) for both MILI (26–27 nt) and MIWI piRNAs (29–30 nt). (H) Comparison of genomic origins for MVH-associated piRNAs. Genotype of donor animals indicated (one wild-type and triplicate *Mvh*^+/KI^ mutant samples). The pie charts below show that most of the piRNAs associated with MVH come from non-annotated genomic regions, with the majority of them originating from previously described top 50 pachytene piRNA clusters. See also Table S2. (I) Mapping of piRNA 5′ ends and sorting into groups based on a preferential association with MILI or MIWI, or their exclusive presence in MVH libraries. The majority of MVH piRNAs are those associated with both PIWI proteins, but more similar to those found in MIWI complexes. (J) Pearson correlation coefficient (PCC) was calculated for the abundance of individual piRNAs sharing the same 5′ ends and is shown as PCC^∗^100. MVH mutation does not affect distribution and abundance of MILI and MIWI piRNAs, and MVH piRNA abundance correlates better with MIWI piRNAs than those of MILI piRNAs. (K) Apart from piRNAs, long reads of 51 nt (corresponds to maximum sequencing length; black arrowhead) were also found in MVH complexes. Genome annotations for these are displayed. While the origin of the mutant (*Mvh*^*+*/*KI*^) long reads resembles that of pachytene piRNAs, the 51-mers of the wild-type (*Mvh*^*+*/*+*^) preferentially come from repeats. The proportion of non-annotated reads mapping to the top pachytene piRNA clusters is shown in the pie charts. (L) The plots show the 5′ end distance between targeting piRNAs and produced piRNAs or MVH-bound 51-mers (MVH long reads) calculated over 500 cellular transcripts that are the top targets for antisense piRNAs. The enrichment of 9-nt distance (the ping-pong signature) is apparent in between the MILI and MIWI piRNAs, as well as between these piRNAs and 51-mers associated with MVH. See also Figure S5.

The *Mvh*^*+*/*KI*^ mutant reveals a delayed spermatogenic arrest when compared with that seen in the *Mvh*^*−*/*KI*^ mutant. Germ cells in the *Mvh*^*−*/*KI*^ mutant do not proceed beyond meiotic pachytene spermatocytes (Figures 2D–2F), while cells in the *Mvh*^*+*/*KI*^ mutant testes complete meiosis and arrest uniformly at post-meiotic haploid round spermatids (Figures 4A and S4A). These arrested mutant germ cells are probably eliminated by apoptosis (Figure S4B), as mature sperm is not detected in the epididymis of the *Mvh*^*+*/*KI*^ mutant (Figure 4B). Periodic acid-Schiff staining of adult testes sections identifies the developing acrosomal vesicle in round spermatids of both wild-type and *Mvh*^*+*/*KI*^ mutants (Figure 4C). However, the *Mvh*^*+*/*KI*^ mutant round spermatids fail to enter spermiogenesis, the post-meiotic cellular differentiation, and chromatin condensation events that are needed to form mature sperm. In the analyses presented above (Figures 2G–2I), we demonstrated a lack of transposon derepression in multiple biological replicates of the *Mvh*^*+*/*KI*^ mutant. We confirmed this conclusion by examining additional *Mvh*^*+*/*KI*^ mutant mice by western analysis for L1ORF1p in testicular lysates (Figure 4D). Although aged animals (P120) revealed some expression of L1ORF1p, this is not to the level seen in the *Mvh*^*−*/*KI*^ mutant. We conclude that the late-spermatogenic arrest in the *Mvh*^*+*/*KI*^ mutant is independent of transposon dysregulation.

### MVH Mutant Traps Complexes Containing PIWI Proteins, Pachytene piRNAs, and Slicer Products

Why do round spermatids in the *Mvh*^*+*/*KI*^ mutant fail to proceed to spermiogenesis? Immunofluorescence analysis of purified round spermatids indicates unchanged localization of MVH in the singular large perinuclear RNA-protein granule called chromatoid body (Figures S4C and S4D). Other components of this granule, such as MIWI and MILI, are also properly localized in *Mvh*^*+*/*KI*^ mutant (Figure S4D). Thus, the mutant protein exists in the same subcellular compartment as the wild-type protein, allowing it to interact with its normal RNA/protein partners. The DEAD → DQAD mutation in *Bombyx* Vasa is shown to reduce its in vivo dynamics, and results in entrapment of the mutant protein in stalled piRNA-PIWI complexes (Nishida et al., 2015, Xiol et al., 2014). We examined this possibility by performing anti-MVH immunoprecipitations and subjecting the isolated complexes to mass spectrometry. MVH complexes from adult wild-type testes reveal MIWI, MILI, and TDRD1 as the top interaction partners (Figure 4E). Interestingly, the same components also figure in the complexes isolated from two biological replicates of the *Mvh*^*+*/*KI*^ mutant testes, but show enrichment (Figure 4E). This enrichment of PIWI proteins is confirmed by the presence of prominent 24- to 30-nt small RNA species (as revealed by 5′ end labeling) in MVH complexes from the *Mvh*^*+*/*KI*^ mutant (Figures 4F and S4E). We propose that the catalytic-dead mutation in MVH reduces its in vivo dynamics, creating a stalled complex, preventing disengagement of its normal (transient) interaction partners, similar to what was observed for *Bombyx* Vasa (Xiol et al., 2014).

To identify these MVH-associated small RNAs from the *Mvh*^*+*/*KI*^ mutant, we subjected them to deep-sequencing analysis (triplicate biological samples). For comparison, we prepared libraries from isolated MIWI and MILI complexes from the adult *Mvh*^*+*/*KI*^ mutant (Figure S4E). As controls, similar libraries were also prepared from adult wild-type or *Mvh*^*+*/*−*^ animals. Read-length distribution in the MVH libraries identify the small RNAs to be piRNAs that are normally found in MILI and MIWI complexes, with ∼30 nt of MIWI-bound piRNAs being more abundant (Figure 4G). Genome annotations of the MVH-bound piRNAs are consistent with the origin of a majority of these sequences from intergenic unannotated genomic regions, and ∼80% of these arise from the top 50 pachytene piRNA clusters (Reuter et al., 2011) (Figure 4H and Table S2). This identifies MVH-bound sequences as bona fide pachytene piRNAs. To understand their origin, we identified genomic coordinates of piRNA 5′ ends and compared the MVH-bound sequences with those present in isolated PIWI complexes. This revealed that the majority (∼80%) of MVH-bound piRNAs are also found in either MIWI, MILI, or both (Figure 4I), with the MVH profile highly similar to the one found in MIWI complexes (Figure 4J). Notably, there is striking similarity between the piRNA profiles found in wild-type and *Mvh*^*+*/*KI*^ mutant animals (Figures 4J and S5D). This suggests that the distribution and abundance of pachytene piRNAs bound to MILI and MIWI are not affected in the *Mvh*^*+*/*KI*^ mutant.

Interestingly, the MVH libraries also contained longer reads of 51 nt, which represents the maximum sequenced length of longer RNAs present in the complex (Figure 4K). Genome annotations show that a large proportion of these in the *Mvh*^*+*/*KI*^ mutant arises from intergenic regions, and ∼65% of the sequences map to the top 50 pachytene piRNA clusters (Figure 4K). Approximately 20% of the 51-mers are pre-piRNA intermediates, as they share the same 5′ ends as pachytene piRNAs (Figure 4I). Next, we examined the 51-mers that arise from genic regions. Since pachytene piRNAs are shown to regulate cellular mRNAs by slicing (Goh et al., 2015, Zhang et al., 2015), we examined whether some of the 51-mers could be products of slicer activity on mRNAs (STAR Methods). This reveals that many of the 51-mers could indeed be slicer cleavage products generated by pachytene piRNA-guided MIWI or MILI slicing of genic mRNAs (Figures 4L and S5E) or L1 transposon transcripts (Figure S5F). Given the lack of pachytene piRNA biogenesis defect, we propose that the infertility observed in the *Mvh*^*+*/*KI*^ mutant is likely due to formation of stalled MVH complexes, preventing normal function of pachytene piRNAs in promoting spermiogenesis.

### TDRD9 Is an ATPase, and Its Activity Is Essential for Male Fertility and Transposon Silencing

**Figure 5:**
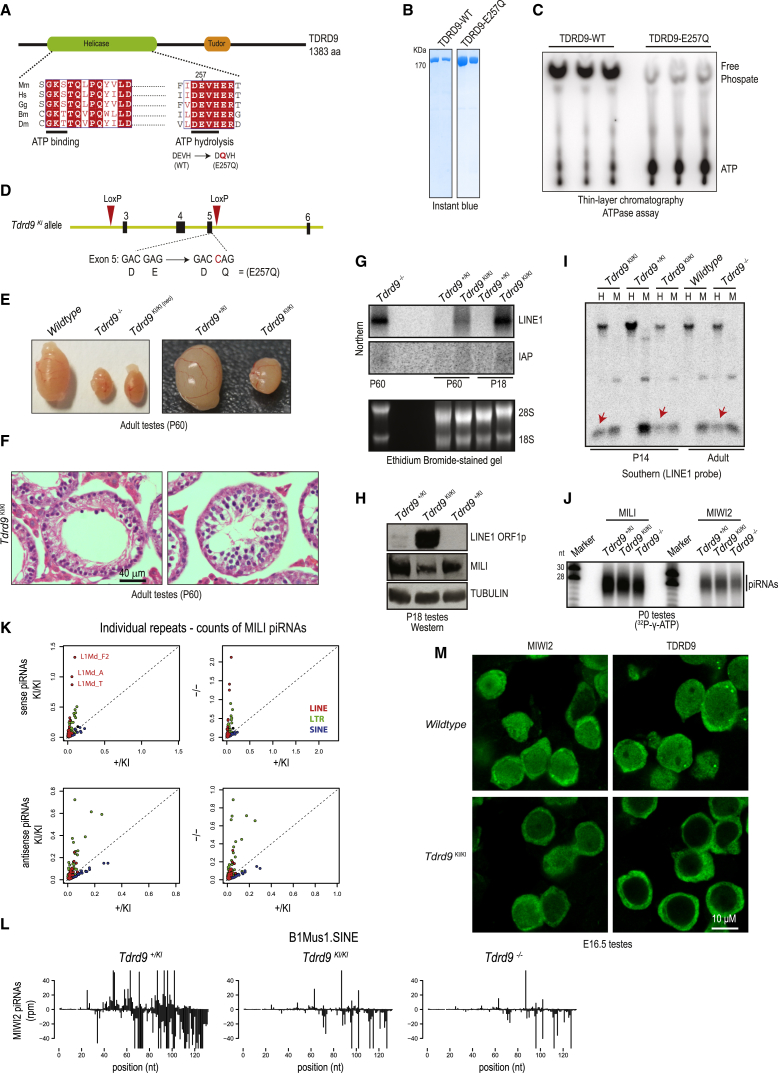
Mouse TDRD9 Is an ATPase, and Its Activity Is Essential for Transposon Silencing, but Not for piRNA Biogenesis (A) Domain architecture of mouse TDRD9 with putative consensus amino acid residues responsible for ATP binding and ATP hydrolysis is shown. The point mutation E257Q that abolishes ATPase activity is indicated. (B) Quality of recombinant mouse TDRD9 protein used for ATPase assays. Wild-type and E257Q point mutant versions were produced. (C) Thin-layer chromatography of ATPase reactions revealing the faster-migrating free phosphate in the presence of the wild-type TDRD9 protein. (D) Creation of the catalytic-dead *Tdrd9* knockin (KI) mouse carrying the E257Q mutation in the ATPase motif (DEVH → DQVH). The same mouse line also allows creation of the knockout (−/−) mutant, by using *loxP* sites flanking exons 3–5. See also Figure S6. (E) Representative image of adult testes from indicated genotypes, showing atrophied testes in homozygous *Tdrd9* knockout and knockin mutants. (F) H&E staining of adult testes from homozygous *Tdrd9* knockin mutant showing arrested germ cell development. Scale bar, 40 μm. See also Figure S7A. (G) Northern analysis of transposons in total testicular RNA, showing derepression of LINE1 retrotransposons in homozygous *Tdrd9* knockout and knockin mutants. Age of donor animals is indicated. (H) Western analysis of total testicular lysates for L1ORF1p expression. MILI (germ cell marker) and TUBULIN (loading control) expression was also examined. (I) Methylation-sensitive Southern blotting for LINE1 genomic loci. The red arrows point to fragments appearing under conditions of reduced DNA methylation in the homozygous *Tdrd9* mutants. H, HpaII-digested DNA; M, MspI-digested DNA. (J) Immunoprecipitation of PIWI proteins and 5′ end labeling of associated small RNAs from neonatal (P0) testes. (K) Comparison of MILI-associated piRNAs mapping to individual repeats. There is a striking enrichment of the piRNAs produced from LINE and LTR repeats in *Tdrd9* mutants (*Tdrd9*^*KI*/*KI*^ and *Tdrd9*^*−*/*−*^). See also Figure S8. (L) Graphs show the distribution of MIWI2-associated piRNAs mapped along B1Mus1.SINE consensus sequence, revealing a depletion of piRNAs in the *Tdrd9*^*KI*/*KI*^ and *Tdrd9*^*−*/*−*^ mutants. (M) Immunofluorescence analysis of indicated proteins in embryonic testes (embryonic day 16.5) of the different genotypes. Note the nucleo-cytoplasmic distribution of TDRD9 in wild-type germ cells, while it is restricted to the cytoplasm in the *Tdrd9*^*KI*/*KI*^ mutant.

Another RNA helicase family member (Figure 5A) implicated in the mammalian piRNA pathway is TDRD9 (Shoji et al., 2009). It is shown to interact with MIWI2 and demonstrated to be essential for transposon silencing and male fertility in mice. To directly examine its ATPase activity, we produced recombinant mouse TDRD9 (Figure 5B) and incubated the protein with radioactive [γ-^32^P]ATP (Figure 5C). As visualized by thin-layer chromatography, this resulted in hydrolysis of ATP and liberation of radioactive free phosphate. Confirming that the activity is inherent to the protein, introduction of a single amino acid substitution (E257Q) in the catalytic motif (DEVH → DQVH) abolishes it (Figure 5C).

To examine the in vivo significance of this activity, we created the knockin allele (referred to as *Tdrd9*^*KI*^) with the inactivating mutation (E257Q) within the ATPase motif (DEVH → DQVH) of TDRD9 (Figures 5D and S6). The design of the mouse also allowed us to create a knockout allele (referred to as *Tdrd9*^*−*^). While females display no impact on fertility, homozygous *Tdrd9*^*KI*/*KI*^ males are infertile, indicating an essential role for the ATPase activity of TDRD9 in spermatogenesis. Our homozygous knockout mutant males are also infertile, similar to what is reported for an independent knockout allele generated previously (Shoji et al., 2009). Visual inspection of the *Tdrd9*^*KI*/*KI*^ mutant testes reveals a highly atrophied tissue that is very similar to that from *Tdrd9*^*−*/*−*^ mice (Figure 5E). Histological analyses of adult testes sections from the *Tdrd9*^*KI*/*KI*^ mutant show the presence of narrow seminiferous tubules that appear empty and are devoid of maturing germ cells (Figures 5F and S7A). Arrested germ cells in the *Tdrd9*^*KI*/*KI*^ mutant lack the γ-H2AX-positive XY body (Figure S7B) and undergo apoptosis (Figure S7C), leading to an absence of pachytene spermatocytes.

Examination of transposon transcript levels reveals derepression of the LINE1 transcripts in *Tdrd9*^*KI*/*KI*^ mutants, similar to that seen in the knockout animals (Figure 5G). Notably, IAP levels are not elevated in both homozygous *Tdrd9* knockout and knockin mutants. The LINE1 transcripts are translated, as the L1ORF1p is detected in testes lysates (Figure 5H) or within mutant germ cells in testes sections (Figure S7D). Loss of DNA methylation on LINE1 genomic copies is responsible for L1 activation in the *Tdrd9*^*KI*/*KI*^ mutant (Figure 5I). These data indicate that the ATPase activity of TDRD9 is essential for proper progression of spermatogenesis and transposon suppression in mice.

### ATPase Activity of TDRD9 Is Dispensable for piRNA Biogenesis but Required for Its Nuclear Accumulation

Given the transposon derepression and loss of DNA methylation in the *Tdrd9* mutant, we examined the status of embryonic piRNA biogenesis using P0 testes. Isolated MILI and MIWI2 complexes were examined for association with piRNAs, which revealed no difference in the *Tdrd9*^*KI*/*KI*^ and *Tdrd9*^*−*/*−*^ mutants when compared with heterozygous *Tdrd9*^*+*/*KI*^ animals (Figure 5J). Deep-sequencing analysis reveals expected read-length distributions of ∼26-nt MILI piRNAs and ∼28-nt MIWI2 piRNAs in the libraries (Figure S8A). Genome mapping indicates that piRNAs arising from LINE1 and IAP reads are present in the mutants. In fact, there is even an increase in their levels in the *Tdrd9*^*KI*/*KI*^ and *Tdrd9*^*−*/*−*^ mutants (Figures 5K, S8B, and S8C), also previously noted in an independent knockout mutant (Shoji et al., 2009). As mainly the levels of L1 sense piRNAs are elevated, this may just be the consequence of the increased abundance of substrates due to transposon derepression, which are now processed into piRNAs. In contrast, reads mapping to SINE elements were depleted in the *Tdrd9*^*KI*/*KI*^ and *Tdrd9*^*−*/*−*^ mutants (Figures 5L and S8D). A similar loss of SINE piRNAs was noted in the previous study that examined a *Tdrd9* knockout mutant, but SINE element DNA methylation was unaffected (Shoji et al., 2009). As expected with the proper loading of MIWI2, the protein is nuclear in the *Tdrd9*^*KI*/*KI*^ mutant (Figure 5M), indicating that any defect in the mutant is downstream of piRNA biogenesis. To our surprise TDRD9, which is normally distributed in both the nucleus and cytoplasm, appears exclusively cytosolic in the *Tdrd9*^*KI*/*KI*^ mutant cells (Figure 5M). This indicates that the ATPase activity of TDRD9 is essential for its nuclear accumulation and its potential role in transcriptional silencing of transposable elements, identifying the TDRD9 as a nuclear effector.

## Discussion

The piRNA pathway operates in both the nucleus and cytoplasm of germ cells. In the mouse embryonic male germline, coordinated action of cytoplasmic MILI and nuclear MIWI2 ensures that transposons remain silenced. Here we investigated a piRNA biogenesis pathway that uses slicing by cytosolic MILI to identify target transcripts as piRNA precursors. This leads to loading of target-derived piRNAs into both MIWI2 and MILI. After cytoplasmic loading, MIWI2 is licensed to enter the nucleus where it carries out transcriptional silencing activities, while MILI surveys the cytoplasmic space.

**Figure 6:**
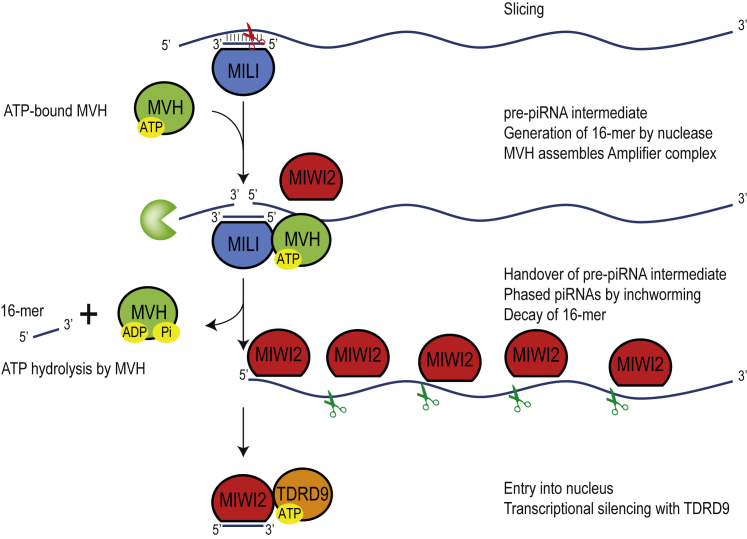
Distinct Roles of RNA Helicases in the Nuclear piRNA Pathway in the Mouse Male Germline A model tracing the current knowledge of MILI slicing-triggered piRNA biogenesis in mice. Slicing of a target RNA creates two cleavage fragments with distinct fates. One of the fragments becomes a substrate for production of a series of phased piRNAs that associate with MIWI2 (and also MILI, not shown in the model). The second fragment is discarded for degradation, but a part of it that is protected by the base-paired piRNA-MILI complex is recovered as a 16-mer by-product in MILI complexes. Entry of the piRNA-producing cleavage fragment into the biogenesis machinery depends on ATPase activity of MVH. In contrast, ATPase activity of TDRD9 is exclusively required for the transposon silencing function in the nucleus.

Endonucleolytic cleavage (slicing) of a target by MILI generates two cleavage fragments with very distinct fates. One of the cleavage fragments (the one carrying the 5′ monophosphate at the site of cleavage) is destined to become a piRNA precursor (represented by the 51-nt pre-piRNA intermediate), while the second fragment is processed into a 16-nt by-product and eventually eliminated (Figures 1 and 6). We demonstrated that the ATPase activity of the RNA helicase MVH is essential for the pre-piRNA intermediate to mature as piRNAs. Mice lacking catalytically active MVH are still able to generate MILI slicer products, but these fail to be used for piRNA production (Figure 3I). Once the cleavage fragment enters the piRNA biogenesis machinery, a series of non-overlapping piRNAs are generated from it, the first one being a secondary piRNA whose 5′ end is same as that of the cleavage fragment, followed by a series of non-overlapping/phased inchworm or trail piRNAs (Han et al., 2015, Mohn et al., 2015, Yang et al., 2016), generated by a process we termed inchworming (Yang et al., 2016). We propose that absence of catalytic activity in MVH causes a failure to hand over the pre-piRNA intermediate to the biogenesis machinery, leading to a loss of all the phased piRNAs triggered by MILI slicing. Although MIWI2 is the main beneficiary of such a biogenesis pathway, MILI also receives some of these target-derived sequences. The slicing-triggered piRNA biogenesis pathway is of paramount importance for L1 retrotransposon silencing, as a majority of MILI-bound piRNAs in the mouse embryonic germline have a sense orientation to the repeat element (Figure S3F). Hence, piRNAs triggered by MILI slicing will be essentially antisense to these elements, and hence functional in post-transcriptional and transcriptional silencing by MILI and MIWI2, respectively.

Studies in *B. mori* (silkworm) BmN4 cells have shown that insect Vasa facilitates a similar handover of the slicer cleavage fragment from one PIWI protein to another, within a complex that we termed the Amplifier (Xiol et al., 2014). It is likely that a similar complex containing MVH, MILI, and MIWI2 is assembled in the mouse embryonic germline. Such a complex will ensure that a PIWI protein is available in the vicinity for immediately receiving the cleavage fragment, which otherwise would be rapidly degraded by cytoplasmic ribonucleases. It is also shown that slicer products of PIWI slicing do not easily dissociate from the PIWI protein and require the ATPase-driven unwinding activity of insect Vasa to aid in this process (Nishida et al., 2015), a possibility that is also supported by the recent structural information on the silkworm PIWI protein Siwi (Matsumoto et al., 2016). This slow rate of slicer-generated product release might explain the formation of the 16-mer by-products, as this requires the non-productive cleavage fragment to be present within the PIWI complex. In addition to the reporter-derived 16-mers (Figure 1), we can identify such by-products of MILI slicing from endogenous L1 targets in mouse small RNA libraries (Figures S3G and S3H). We note that a 19-mer fragment that is complementary to pachytene piRNAs is reported (Berninger et al., 2011), and probably is a by-product generated by the larger footprint of the MIWI slicer (Reuter et al., 2011). Similar 16-mer by-products are also generated by insect PIWI proteins in *Bombyx* BmN4 cells (Xiol et al., 2012) and *Drosophila* ovaries (Wang et al., 2015). Finally, in addition to MVH (Kuramochi-Miyagawa et al., 2010), a number of other factors such as the TDRD12-EXD1 complex (Pandey et al., 2013, Yang et al., 2016), FKBP6 (Xiol et al., 2012), and TDRD1 (Kojima et al., 2009, Reuter et al., 2009) are also required for generation of MIWI2 piRNAs. How these factors collaborate together with the Amplifier complex remains to be seen, and would require a combination of biochemical and structural investigations.

The dominant-negative effect of the ATPase mutation in MVH reveals a poorly appreciated role for MVH in the adult germline. Pachytene piRNAs in MILI and MIWI are shown to target complementary transposon and cellular mRNAs for slicing (Goh et al., 2015, Reuter et al., 2011, Zhang et al., 2015). Partial complementarity base pairing is also shown to be used for targeting cellular transcripts for degradation by recruitment of the deadenylation machinery (Gou et al., 2014). This raises the possibility that pachytene piRNAs might recognize thousands of transcripts and tag them for elimination during spermiogenesis, a process that progressively leads to emptying of cytosolic contents and condensation of the chromatin. Our finding that the dominant-negative ATPase mutant MVH traps PIWI proteins MILI and MIWI (Figure 4E), pachytene piRNAs (Figure 4F), and slicer products of transposon and genic mRNAs (Figures 4L and S5F) is suggestive of a potential role for MVH in their function. We note that slicer-inactive mutant MIWI protein that fails to cleave target transcripts also shows such a dominant-negative effect in heterozygous mice carrying both the wild-type and knockin alleles (Reuter et al., 2011). We propose that the dominant-negative effect of the catalytic-dead mutation in MVH is likely due to a combination of effects, including the failure to eliminate/process slicer cleavage fragments and creation of stalled inactive complexes containing the mutant MVH, PIWI proteins, and piRNAs, as we demonstrated (Figures 4E and 4F).

The TDRD9 ortholog in insects, Spn-E, is described as a piRNA biogenesis factor, and depends on its ATPase activity for ensuring piRNA biogenesis and fertility (Malone et al., 2009, Nishida et al., 2015). However, study of the mouse ortholog already indicated that piRNA biogenesis is not affected by loss of TDRD9 (Shoji et al., 2009). It was also shown that nuclear accumulation of MIWI2 was unaffected in the *Tdrd9* knockout. We now examined by high-throughput sequencing the state of embryonic piRNA biogenesis in mice expressing catalytic-dead TDRD9 and revealed no changes that can explain transposon derepression. We biochemically demonstrated that TDRD9 is an ATPase and that this activity is essential for transposon silencing and male fertility, positioning TDRD9 as a nuclear effector protein in the mammalian piRNA pathway.

## STAR★Methods

### Key Resources Table

**Table:** 

REAGENT or RESOURCE	SOURCE	IDENTIFIER
**Antibodies**		

Rabbit polyclonal anti-MVH	Abcam	ab13840; RRID: AB_443012
Rabbit polyclonal anti γ-H2AX	Abcam	ab2893; RRID: AB_303388
Rabbit polyclonal anti-β-TUBULIN	Abcam	ab6046; RRID: AB_2210370
Rabbit polyclonal anti-ACTIN (I-19)	Santa Cruz Biotechnology	sc-1616-R; RRID: AB_630836
Mouse monoclonal anti-MILI (13E3)	(Reuter et al., 2009)	N/A
Rabbit polyclonal anti-MILI	(Reuter et al., 2009)	N/A
Rabbit polyclonal anti-MIWI	(Reuter et al., 2011)	N/A
Rabbit polyclonal anti-MIWI2	(Pandey et al., 2013)	N/A
Rabbit polyclonal anti-LINE1 ORF1p	This study	N/A
Rabbit polyclonal anti-TDRD9	(Shoji et al., 2009)	N/A

**Bacterial and Virus Strains**		

DH10EMBacY bacterial strain	(Bieniossek et al., 2012)	N/A

**Chemicals, Peptides, and Recombinant Proteins**		

D-desthiobiotin	IBA	2-1000-001
Complete EDTA-free protease inhibitor	Roche	11 873 580 001
Tissue-Tek™ CRYO-OCT Compound	Fisher Scientific	14-373-65
DAPI	Bio-Rad	10043282
Bouin’s solution	Sigma	HT10132
Hematoxylin solution, Harris modified	Sigma	HHS16
Eosin Y solution with phloxine	Sigma	HT110332
Permount	Fisher Scientific	SP15-100
sodium deoxycholate	Sigma	30968
Slowfade Gold Antifade Reagent	Life technologies	S36942

**Critical Commercial Assays**		

Periodic Acid-Schiff Kit	Sigma	395B
NEBNext Multiplex Small RNA Library Prep Set for Illumina	NEB	E7300
MinElute Gel Extraction Kit	Qiagen	28604

**Deposited Data**		

Deep sequencing datasets	This study	GEO: GSE95580

**Experimental Models: Cell Lines**		

Sf21 insect cells for protein production	Eukaryotic Expression Facility, EMBL Grenoble, France	N/A
High Five insect cells for protein production	Eukaryotic Expression Facility, EMBL Grenoble, France	N/A
A9 mouse ES cells; 129P2/OlaHsd x C57BL/6J	Transgenic Mouse Facility, EMBL Monterotondo, Italy	N/A

**Experimental Models: Organisms/Strains**		

Mouse: *Rosa26-pi* knockin	(Yang et al., 2016)	Available from Lead Contact
Mouse: *Mvh*^*E446Q*^ knockin/conditional KO	This study	EMMA accession: EM: 09169
Mouse: *Tdrd9*^*E257Q*^ knockin/conditional KO	This study	EMMA accession: EM: 08489
Mouse: Del-FLPeR transgenic	(Farley et al., 2000)	N/A
Mouse: CMV-Cre	(Schwenk et al., 1995)	N/A

**Oligonucleotides**		

ZY530: Forward Mvh genotyping TAGCAGGAATTTGGAGGCCA	This study	N/A
ZY533: Reverse Mvh genotyping ACCTTCGTTTCTGAGACAGG	This study	N/A
RRoligo428: Forward Tdrd9 WT GACCACTGGAGTCCTGCTTC	This study	N/A
RRoligo429: Reverse Tdrd9 WT GCCCAGGTTTTGAACCCTAT	This study	N/A
RRoligo431: Reverse Tdrd9 KIneo GGGGAACTTCCTGACTAGGG	This study	N/A
RRoligo 432: Forward Tdrd9 KI CTGGAGCCAGTGTGTGTCAG	This study	N/A

**Recombinant DNA**		

pACEBac2	(Bieniossek et al., 2012)	N/A
Mouse Tdrd9 cDNA	(Shoji et al., 2009)	N/A
pETM-11-Line1ORF1p antigen	This study	N/A
Mouse Mvh cDNA	This study	N/A

**Software and Algorithms**		

Pipeline for small RNA analysis	(Olson et al., 2008)	N/A
Cutadapt		http://dx.doi.org/10.14806/ej.17.1.200
R		https://www.r-project.org
Bowtie	(Langmead et al., 2009)	N/A
Bioconductor		https://www.bioconductor.org/

**Other**		

Chelating Sepharose Fast Flow beads	GE Healthcare	17-0575-01
StrepTrap HP	GE Healthcare	28-9075-46
Superose 6 10/300 GL	GE Healthcare	17-5172-01
TLC PEI Cellulose F	Merck	105579
Micro-Spin G25 columnS	GE Healthcare	27-5325-01
MethaPhor agarose	Lonza	50180

### Contact for Reagent and Resource Sharing

Further information and requests for resources and reagents should be directed to and will be fulfilled by the Lead Contact, Ramesh S. Pillai (ramesh.pillai@unige.ch).

### Experimental Model and Subject Details

#### Animal Work

Mutant mice were generated at the Transgenic Mouse Facility of European Molecular Biology Laboratory (EMBL) Monterotondo, Italy. The animal facilities are operated according to international animal welfare rules (Federation for Laboratory Animal Science Associations guidelines and recommendations). Founder mice were shipped to EMBL Grenoble, France, where they were housed in the animal facility of Commissariat à l’énergie atomique et aux énergies alternatives (CEA), Grenoble. Experiments in Grenoble, France were covered by an authorization (no. 381007) from the Direction Departementale de la Protection des Populations, Prefecture de l’Isere. After the Pillai lab moved to Switzerland, some of the experiments were carried out in the Animal Facility of Sciences III, University of Geneva. All Geneva experiments were conducted with authorization (no. GE/102/16) from the Republic and Canton of Geneva.

#### *Mvh*^*E446Q*^ Knockin and *Mvh* Knockout Mice

The *Mvh* (also known as *Ddx4*) gene locus is located on mouse chromosome 13 and consists of 22 exons (Figures S2A and S2B). We targeted the endogenous *Mvh* locus of the hybrid 129P2/OlaHsd x C57BL/6J male embryonic stem (ES) cell line A9 using a targeting vector that replaces a genomic region encompassing the exons 15-16. This enabled introduction of a single nucleotide mutation in the exon 16 that changes the encoded amino acid (E446Q) within the helicase catalytic motif (DEAD →DQAD), and *loxP* sites flanking this exon 16, allowing generation of a knockout allele. Selection of the targeted ES cells was achieved by the introduction of a Neomycin (*neo*) selection marker into the 15^th^ intron, which can also be removed later in the mice using the flanking FLP recombinase target (*FRT*) sites.

Electroporated A9 ES cells were selected (Neomycin or G418) and 300 clones screened by Southern blotting with probes recognizing the 5′ (XbaI-digested DNA) and 3′ (HindIII-digested DNA) regions flanking the targeted site (Figure S2C). Only 3 ES cell clones were positively identified by both probes to be properly targeted. These ES cell clones were karyotyped for genome integrity. One clone was selected for injection into C57BL/6N host embryos (8-cell stage) for mouse generation. One founder male was obtained and identified by coat color (agouti) and genotyped by PCR of tail genomic DNA for presence of a targeting construct-specific sequence. Backcrosses with C57BL/6J Rj (Janvier labs) wild-type females were performed to transfer the mutation to the C57BL/6 background to obtain heterozygous mice carrying the targeted allele *Mvh*^*E446Q*(*neo*)^, that still carried the Neomycin cassette.

Heterozygous females were crossed with Del-FLPeR (flipper) male mice (Farley et al., 2000) to remove the PGK-Keo (kanamycin/neomycin)-polyA cassette to generate the *Mvh*^*E446Q*^ knockin allele (hereafter referred to as *Mvh*^*KI*^). Male mice carrying the knockin allele in all contexts are infertile. To generate the heterozygous *Mvh*-null mice (*Mvh*^*+*/*−*^), the *Mvh*^*KI*^ females were crossed with male transgenic mice ubiquitously expressing the X-linked CMV-Cre recombinase (Schwenk et al., 1995). This leads to deletion of exon 16 in *Mvh* gene locus, creating the knockout allele. Intercrosses generated the following experimental mice: *Mvh*^*+*/*−*^, *Mvh*^*+*/*KI*^, *Mvh*^*−*/*KI*^ and *Mvh*^−/−^. Only male mice were used for experiments, with the age of the donor mice being indicated in the text and relevant figures. Female mice carrying the *Mvh* knockout and knockin alleles were further crossed with the *Rosa26-pi* reporter male mice (Yang et al., 2016) to bring the artificial piRNA reporter into the different *Mvh* genetic backgrounds. Briefly, the artificial piRNA reporter had a DsRed coding sequence with a 3′-UTR based on noncoding (all ATGs were mutated) LacZ sequence. The 3′-UTR contains 35 perfectly complementary binding sites for different MILI-bound piRNAs that are abundantly expressed in the embryonic mouse male germline. (Yang et al., 2016). We previously demonstrated that this reporter is a target for MILI slicing for generation of piRNAs. New born pups (P0) and adults were collected for piRNA analysis. Mutant mice (*Mvh*^*E446Q*^) are deposited with the European Mouse Mutant Archive (EMMA) under the accession number EM:09169.

##### Genotyping

Primers to detect bands (Figure S2D) corresponding to the wild-type (859 bp), knockin (1102 bp) and knockout (444 bp) alleles were ZY530 (TAGCAGGAATTTGGAGGCCA) and ZY533 (ACCTTCGTTTCTGAGACAGG).

#### *Tdrd9*^*E257Q*^ Knockin and *Tdrd9* Knockout Mice

The *Tdrd9* gene locus is located on mouse chromosome 12 and is composed of 36 exons, with the translated sequence being provided by exons 1-36 (Figure S6A). The N-terminal RNA helicase domain is contributed by exons 3-18, while the C-terminal tudor domain is encoded by exons 25-28. The *Tdrd9* locus in the hybrid 129P2/OlaHsd x C57BL/6J male embryonic stem (ES) cell line A9 was targeted using a genomic fragment that replaced exons 3-8. The replacement cassette carried a single point mutation in exon 5, creating a point mutation (E257Q) in the ATPase motif (DEVH→DQVH). In addition, *loxP* sites were placed flanking exons 3-5, allowing the production of knockout mutant animals. To enable selection of the targeted ES cells, the targeting construct also brought in the PGK-Keo (kanamycin-neomycin)-polyA cassette in the intron immediately downstream of exon 5. The selection cassette was flanked by *FRT* sites to allow for excision from the genome.

Electroporated A9 ES cells were selected (neomycin or G418) and 300 clones screened by Southern blotting with probes recognizing the 5′ (BamHI-digested DNA) and 3′ (AseI-digested DNA) regions flanking the targeted site. Only 8 ES cell clones were positively identified by both probes to be properly targeted (Figure S6B). These ES cell clones were karyotyped for genome integrity. One clone (#5D) was used for injection into C57BL/6N host embryos (8-cell stage) for mouse generation. One founder animal was obtained and identified by coat color (agouti) and genotyped by PCR of tail genomic DNA for presence of a targeting-construct specific sequence. Backcrosses with C57BL/6J Rj (Janvier labs) wild-type females were performed to transfer the mutation to the C57BL/6 background to obtain heterozygous mice carrying the targeted allele *Tdrd9*^*E257Q*(*neo*)^, still containing the neomycin selection marker. Heterozygous males were crossed with Del-FLPeR (flipper) (Farley et al., 2000) female mice to remove the PGK-Keo cassette to generate the *Tdrd9*^*E257Q*^ knockin allele (hereafter referred to as *Tdrd9*^*KI*^). Heterozygous *Tdrd9* null mice (hereafter referred to as *Tdrd9*^*+*/*−*^) were generated by crossing the *Tdrd9*^*KI*^ females with male transgenic mice expressing the X-linked CMV-Cre recombinase (Schwenk et al., 1995). This leads to deletion of exons 3 to 5, potentially leading to a frame shift and a stop codon in the exon 6. Intercrosses generated the following experimental mice: *Tdrd9*^*+*/*−*^, *Tdrd9*^*+*/*KI*^, *Tdrd9*^*KI*/*KI*^ and *Tdrd9*^−/−^. Only males were used for all experiments and the age of the donor animals is indicated in the text and relevant figures. Mutant mice (*Tdrd9*^*E257Q*^) are deposited with the European Mouse Mutant Archive (EMMA) under the accession number EM:08489.

##### Genotyping

Tails of experimental mice were digested in 500 μl tail buffer (50 mM Tris-HCl, pH 8.0, 100 mM EDTA, 100 mM NaCl, 1% SDS) with 2.5 μg of Proteinase K at 55°C overnight. After spinning at 16000 × g for 10 min to remove hairs, supernatants were transferred into a new tube and the DNA was precipitated by adding 500 μl of isopropanol. Samples were spun in the centrifuge at 16000 × g for 10 min, and the resulting pellet was washed with 1 ml 70% Ethanol. The pellet was dried and resuspended for at least 1 hour at 37°C in 100 to 150 μl of 10 mM Tris-HCl, pH 8.0. Approximately, 1-1.5 μl of genomic DNA were used for PCR.

Primers to detect bands (Figure S6C) corresponding to the wild-type (494 bp) or knockin (642 bp) *Tdrd9* alleles were RRoligo428 (GACCACTGGAGTCCTGCTTC) and RRoligo429 (GCCCAGGTTTTGAACCCTAT). Primers to detect the *Tdrd9*^*KI*(Neo)^ allele (632 bp) were RRoligo428 (GACCACTGGAGTCCTGCTTC) and RRoligo431 (GGGGAACTTCCTGACTAGGG). Primers to detect the *Tdrd9*^*−*(Neo)^ allele (403 bp) were RRoligo 432 (CTGGAGCCAGTGTGTGTCAG) and RRoligo431 (GGGGAACTTCCTGACTAGGG).

Reaction mix for 25 μl PCR reactions: 1 × Taq buffer (without MgCl_2_), 2 mM MgCl_2_, 0.5 μl dNTPs mix (stock 10 mM), 0.5 μl primer mix (stock 20 nM each), 1.0 μl tail DNA (100-200 ng), 0.5 μl Taq Pol (EMBL Protein Expression Facility, Heidelberg), water to make 25 μl final volume. Reactions for oligo pair 428 + 429 were run using the following conditions (94°C, 20 sec; 57°C, 30 sec; 72°C, 30 sec) for 35 cycles. Reactions for oligo pair 428 + 431+ 432 were run using the following conditions (94°C, 20 sec; 63°C, 40 sec; 72°C, 30 sec) for 35 cycles.

### Method Details

#### Clones and Constructs

The full-length complementary DNA (cDNA) for *Mouse Vasa Homolog* (MVH; 1-728 aa; GenBank Accession no. NM_001145885) was isolated by reverse-transcription PCR (RT-PCR) from mouse testis total RNA. The cDNA for mouse *Tdrd9* (1383 aa; GenBank Accession no. AB362563) was a kind gift of Dr. Shinichiro Chuma (Shoji et al., 2009). For the production of full-length TDRD9 protein we used eukaryotic expression systems based on insect ovary-derived cells: *Spodoptera frugiperda* 21 (*Sf*21) or the *Trichoplusia ni* High Five cells. Full-length coding sequence for mouse TDRD9 (1-1383 aa) and TDRD9^E257Q^ ATPase mutant, which has a single amino acid mutation E257Q in the catalytic motif (DEVH→DQVH), were cloned into the NheI and KpnI restriction sites of the vector pACEBac2 to express the recombinant proteins as 6xHis-Sumo-StrepIII- fusions. For expression of an antigen to raise antibodies against the LINE1 transposon gene product, LINE1 ORF1p, sequences encoding the following sequence was cloned into the bacterial expression vector pETM-11 (6xHis-tag fusions).

##### Sequence of the L1ORF1p Antigen

MKHHHHHHPMSDYDIPTTENLYFQGAMATGGEQMGRDPNSSIPGSLVPTSFRDYQMAKGKRKNPTNRNQDHSPSSERSTPTPPSPGHPNTTENLDPDLKTFLMMMIEDIKKDFHKSLKDLQESTAKELQALKEKQENTAKQVMEMNKTILELKGEVDTIKKTQSEATLEIETLGKRSGTIDASISNRIQEMEERISGAEDSIENIDTTVKENTKCKRILTQNIQVIQDTMRRPNLRIIGIDENEDFQLKGPANIFNKIIEENFPNIKKEMPMIIQEAYRTPNRLDQKRNSSRHIIIRTTNALNKDRILKAVKGERSSN^∗^

#### Antibodies

##### Commercial Antibodies

The following antibodies were purchased: anti-MVH (Abcam, ab13840), anti γ-H2AX (Abcam ab2893), anti-β-TUBULIN (Abcam; ab6046) and anti-ACTIN (Santa Cruz Biotechnology (I-19)-R, sc-1616-R) to detect mouse proteins. For immunofluorescence studies the following secondary antibodies were used: anti-rabbit (Life Technologies; Alexa Fluor 488, A11034), anti-rabbit (Life Technologies; Alexa Fluor 594, A11037) and anti-mouse (Life Technologies; Alexa Fluor 594, A11005). The following secondary antibodies conjugated to HorseRadish Peroxidase were used for Western analyses: anti-rabbit IgG HRP-linked antibody (GE Healthcare; NA934), anti-mouse IgG HRP-linked (GE Healthcare; NA931).

##### Other Antibodies

Antibodies for mouse MILI (mouse monoclonal 13E3 or rabbit polyclonal FGR9) (Reuter et al., 2009), MIWI2 (rabbit polyclonal FCHE) (Pandey et al., 2013), MIWI (rabbit polyclonal 3BW8 and BTO) (Reuter et al., 2011), and TDRD9 (kind gift of Dr. Shinichiro Chuma) (Shoji et al., 2009) are described previously. Antibody for mouse LINE1 ORF1p (rabbit polyclonal GJAE) was raised against an insoluble antigen produced in *E.coli*.

#### Recombinant TDRD9 Production

Full length mouse TDRD9 was cloned in pACEBac2 as a 6xHis-Sumo-StrepIII-fusion protein and the resulting plasmid was transformed to DH10EMBacY competent cells, where the recombination occurs and the bacmid is formed (Bieniossek et al., 2012). Recombinant baculovirus stocks were generated in Sf21 cells and used for infecting exponential growing High Five cells.

Three to four days after cell proliferation arrest, cell pellets were collected with gentle spin (800 × g for 15 min) and resuspended in lysis buffer (20 mM HEPES pH 8.0, 300 mM NaCl, 5 mM MgCl_2_, 10 mM imidazole, 0.05% Tween-20, 10% glycerol, 5 mM 2-mercaptoethanol) supplemented with protease inhibitor (Roche Complete EDTA-free, cat. no. 11 873 580 001; 1 tablet for 50 ml of lysis buffer), or stored at -80°C until further analysis. After 2 minutes of sonication, lysates were spun down at 21000 rpm for 40 minutes and the proteins were purified by Ni^2+^-affinity chromatography. Ideally, 1 to 2 liters of infected High Five cells (at 500,000 cells/ml) were resuspended in 100 ml lysis buffer and incubated with 7 ml of chelating sepharose fast flow beads (GE Healthcare; cat. No. 17-0575-01) previously bound to nickel. Beads were washed in with 20 ml washing buffer (50 mM imidazole in lysis buffer) and high salt washing buffer (50 mM imidazole, 1 M NaCl in lysis buffer). After a final wash in lysis buffer the protein was eluted in 250 mM imidazole in lysis buffer (15 to 20 ml final volume) and immediately loaded on a Streptactin pre-packed column (StrepTrap HP, GE Healthcare, cat. No. 28-9075-46). After a wash in lysis buffer without imidazole, the protein was eluted in the same buffer with 2.5 mM D-desthiobiotin (IBA; cat. no. 2-1000-001). Attempts to concentrate the protein over 1.4 mg/ml were unsuccessful and to avoid protein aggregation, single eluted fractions (without any concentration step) were loaded on a gel filtration column (Superose 6 10/300 GL, GE Healthcare, cat. No.17-5172-01) and monodisperse fractions were collected and used for ATPase assay.

#### ATPase Assay

ATP hydrolysis reactions (20 μl) containing 5 μg of protein were performed in a buffer containing (20 mM HEPES pH 8.0, 300 mM NaCl, 5 mM MgCl_2_, 0.05% Tween-20, 10% glycerol, 5 mM 2-mercaptoethanol), 1 μl of [γ-^32^P] ATP (3000 Ci/mmol, Perkin Elmer) in absence or presence of 25 nM or 0.25 μM cold ATP. Reactions were incubated at 15°C for 30 min and stopped by addition of 5 μl of formic acid. 2.5 μl were spotted on a thin layer chromatography (TLC) plate (TLC PEI Cellulose F, Merc, cat. No. 105579) and migrated in a migration chamber for 45 min in 0.5 M LiCl, 0.5 M formic acid. Free phosphate can be distinguished from ATP because it migrates faster on the TLC plate. The foil was then dried at room temperature and exposed to Storage Phosphor Screens (GE Healthcare; BAS IP MS 2025 E, cat no. 28-9564-75) and scanned (GE Healthcare; Typhoon FLA 9500 IP, cat no. 29-1885-90).

#### Histology and Immunofluorescence

##### Mouse Testes Sections

Collected testes were washed in PBS and immediately fixed in 10 ml of 2% paraformaldehyde at 4°C for 3 hours on a rotating wheel. Tissues were washed twice in PBS and dehydrated in 15% sucrose in PBS for almost 3 hours (till the testes sink to the bottom of the falcon tube). After a further dehydration step in 30% sucrose overnight, tissues were embedded in home-made cryo-mould filled with Andwin Scientific Tissue-Tek™ CRYO-OCT Compound (Fisher Scientific, cat. No. 14-373-65) and frozen on dry ice. Embedded tissues were sent to Histology service at EMBL, Monterotondo in Italy, where after sectioning, 7 μm tissue sections were mounted on glass slides and stored at -80°C. For immunofluorescence experiments, sections were allowed to dry at room temperature for 30 min and fixed in cold 4% paraformaldehyde in PBS (on ice) for 10 min. Slides were then washed in PBS at RT (2 × 5 min) and once in distilled water (5 min). Next, antigen retrieval was performed with Heat-Induced Epitope Retrieval (HIER). Briefly, slides were submerged in 600 ml of 10 mM Citrate Buffer pH 6.0 and heated in a microwave at full power (600 W) for 20 minutes. Alternatively, slides were immersed in (10 mM Tris-EDTA pH 9.0) and similarly heated in a microwave for 10 min: this antigen retrieval buffer gives a better staining for perinuclear granules. Tissues were allowed to cool down at room temperature for at least 45 minutes, washed in PBS and permeabilized in 0.3% Triton X-100 in PBS at RT for 10 minutes. Slides were washed twice in TBS-0.1% Tween20 (TBS-T) and blocked for 30 minutes at room temperature in a humidified chamber in 5% normal goat serum in TBS-T. Primary antibodies were diluted in blocking buffer at different concentrations (see below) and incubated overnight at 4°C. Next day, slides were washed twice in TBS-T, incubated with secondary antibody (anti-mouse or anti-rabbit conjugated to Alexa 488, or 594 fluorophore) in a humidified chamber for 45-60 minutes (dilution 1:1000). Slides were washed twice in TBS-T and incubated with DAPI (0.5 μg/ml, Bio-Rad, cat. No. 10043282) for 5-15 minutes to counterstain the nuclei. Sections were finally washed twice in TBS-T, once in ddH_2_O and mounted with Slowfade Gold Antifade Reagent (Life technologies, cat. No. S36942). Pictures were taken using Leica TCS SP2 AOBS, inverted confocal microscope or Leica TCS SP8.

Primary antibodies concentrations: purified anti-MILI 1:100, crude serum anti-MIWI2 1:50, crude serum anti-MIWI 1:200, crude serum anti-L1ORF 1:200, purified anti-TDRD9 1:50, anti-MVH (Abcam, ab13840) 1:200, anti- γ-H2AX (Abcam, ab2893) 1:200.

##### Histology of Mouse Testes Sections

To prepare the paraffin sections, the mouse testes were washed in PBS, and fixed in 4% paraformaldehyde overnight at 4°C. After washing in PBS, testes were dehydrated in 70% ethanol and stored in 70% ethanol at 4°C. Alternatively isolated testes and epididymis fragments were fixed in Bouin’s solution (Sigma, cat. No HT10132) overnight at room temperature and subsequently washed 3 times in 50% ethanol and in several changes of 70% ethanol, until no yellow dye could be extracted into solution. Samples were sent to Histology service in EMBL, Monterotondo, Italy where they were further dehydrated in 80%, 90%, 96% and 100% ethanol (90 min for each step), followed by incubation in xylene (3 times 30 min). Xylene was removed and replaced with paraffin, and incubated at 56-58°C. Testes were then transferred into plastic molds (Polysciences mold S-22; NC0397999) filled with paraffin, and paraffin was allowed to become solid at room temperature. The testes sections (∼7 μM thickness) were prepared using microtome and mounted on the Superfrost Plus slides with 10% ethanol. The sections were allowed to stretch at 42°C and then stored at room temperature. For histological analysis, the slides containing the paraffin sections were placed in a glass slide holder filled with xylene (2 × 5 min) to remove the paraffin. For rehydration, the slides were incubated in 100% ethanol, 96% ethanol, 70% ethanol, 50% ethanol and water (2 min for each step). Sections were stained with Hematoxylin solution, Harris modified (Sigma, cat. No. HHS16) for 1-3 min and rinsed in running tap water. To destain the colorant, sections were incubated in acidic alcohol (1% HCl in 70% ethanol) for 5-20 sec and rinsed with water. Then the slides were immersed in bluing solution (ammonium solution) for 15-20 sec and rinsed in tap water. Then, sections were stained with Eosin Y solution with phloxine (Sigma, cat. No. HT110332) for 20 sec to 1 min and washed with water. For dehydration, the sections were incubated in 70% (10-20 sec), 96% (30 s), 100% ethanol (2 min) and xylene (2 × 5 min). Few drops of Permount (Fisher Scientific, cat. No. SP15-100) were deposited on the sections and immediately covered with coverslips.

For acrosome staining rehydrated sections were stained with periodic acid and Schiff reagent and counterstained with hematoxylin using Periodic Acid-Schiff Kit (Sigma, cat. no. 395B) according to manufacturer instructions. Dehydration and mounting was performed as described above. The sections were examined and pictures were taken using widefield (Zeiss Axio Imager Z1 or Axio M2) microscopy.

##### Cell Spread Preparation and Staining

Round spermatids were purified from *Mvh*^*+*/*−*^ and *Mvh*^*+*/*KI*^ adult mice using a BSA gradient method (Pivot-Pajot et al., 2003). Next, the cells were used for cell spreads preparation. Microscopic slides (SuperFrost) were immersed in fixer bath (2%, PFA, 0.05% Triton 100x, pH 8.0-8.5) and dried. Small portion of cell suspension (∼10 μl) was mixed with 20 μl of 100 mM sucrose, placed on the slide and left for 1 h to settle. After this time, slides were quickly rinsed twice in PBS, dried and frozen at -80°C. Immunofluorescence was performed as described above with minor changes: the antigen retrieval step (boiling with sodium citrate) was omitted, permeabilisation step was shortened to 5 min and washings were performed in PBS instead of TBST.

#### Northern Blot

Total RNA was extracted from mouse testes using TRIzol RNA extraction kit (Life technology, cat. No. 15596-026), further purified with double phenol-chloroform treatment, precipitated in ethanol for at least 20 minutes at -20°C and resuspended in milli-Q water. For Northern blotting, 8-10 μg of total RNA were resolved in a 1% agarose gel containing 6.7% formaldehyde (v/v). The quality of the migration was assessed by ethidium bromide staining (Figure 2G) and the RNA was transferred by capillarity to a Nylon membrane (Hybond N+, Amersham) for at least 16 h in in 20 × SSC solution (3 M NaCl, 300 mM sodium citrate). After the transfer, the RNA was UV cross-linked to the membrane using a Stratagene “cross linker” (120 mJ/cm^2^ in auto-crosslinking mode). Pre-hybridization was performed for 1,5-2 h in Church buffer (0.25 M sodium phosphate buffer pH 7.2, 1 mM EDTA, 1% BSA, 7% SDS) at 65°C. Probes were labelled with [α-^32^P]dCTP (3000 Ci/mmol, 10 mCi/ml, Perkin Elmer) using the Random Primed DNA Labeling Kit (Roche, cat No. 1004760001). Briefly, 15-30 ng of gel-purified PCR product was used for random-primer transcription with Klenow fragment and radioactive αP^32^-CTP. The resulting probes were filtered on Micro-Spin G25 columns (GE Healthcare, cat. No. 27-5325-01) to remove unincorporated nucleotides, denatured for 5 min at 95°C and incubated with the membrane in 10 ml Church buffer at 65°C overnight. Next day, washing was performed at 65°C as follows: twice 15 min each with buffer-1 (2× SSC, 0.1% SDS) and twice 15 min each with buffer-2 (0.2× SSC, 0.1% SDS). The membrane was wrapped in Saranfilm, exposed to Storage Phosphor Screens (GE Healthcare; BAS IP MS 2025 E, cat no. 28-9564-75) and scanned (GE Healthcare; Typhoon FLA 9500 IP, cat no. 29-1885-90). The signal of 23S and 18S RNA visualized by ethidium bromide staining was used as a loading control.

The LINE-1 (L1) probe was amplified with primers RP469 (gaagttcccaacatagagtcc) and RP470 (agtgggcagagtattctctgc), on the template of cloned L1 fragment (kindly provided by Donal O’Carroll). The sequence corresponds to 513-1,628 bp of Mouse L1Md-A2 repetitive element (GenBank accession No. M13002.1). IAP probe was similarly prepared using primers JW7 (GGGAATACTAATGTCCCTCG) and JW8 (CAACCAGAATCTTCTACGGC). The sequence corresponds to 3758-5171 bp of Mus musculus retrotransposon IAP (GenBank accession No. EU183301.1).

#### Methylation-sensitive Southern Blot

Genomic DNA from adult mouse testes or from P14 mouse testes were extracted using DNAzol reagent (Life technologies, cat. No. 10503-027), followed by three washes in 70% ethanol and finally resuspended in 8 mM NaOH. Basic pH was neutralized by adding a few microliters of 1 M HEPES pH 8.0. Approximately 5 μg of genomic DNA was digested overnight at 37°C with 20 U of methylation-sensitive restriction enzyme HpaII (New England Biolabs, R0171S) or 40 U of methylation insensitive restriction enzyme MspI (New England Biolabs, R0106S). The reaction buffer included Cut Smart buffer 1 × (New England Biolabs), spermidine 0.1 M, DTT 0.1 M and 0.25 μl of RNaseH in 50 μl final volume. The digested DNA was directly loaded on 1% Agarose gel in 1 × TBE. After run, the gel was incubated in 0.25 M HCl for 15 min and washed in 0.5 M NaOH, 1.5 M NaCl twice (10 min and 45 min). Acidic pH was neutralized by immersing the gel in 1 M Tris-HCl pH 8.0, 1.5 M NaCl for 20 minutes. The gel was finally washed with water and soaked in 20 × SSC buffer. DNA was transferred via passive capillary blotting overnight onto a Nylon membrane (Amersham, Hybond N+) using 20 × SSC buffer. The next day the membrane was UV cross-linked (Stratagene; Stratalinker, 1200 μJx100) using the auto-crosslinking mode. Membrane was washed in 2 × SSC and immediately put in 65°C pre-warmed church buffer for 2 hours. LINE-1 probe was prepared as described in the previous section and incubated with the membrane overnight. The following day the membrane was washed in 2 × SSC, 0.1% SDS (twice for 15 minutes) and in 0.2 × SSC, 0.1% SDS (twice for 15 minutes). Finally, the membrane was exposed to Storage Phosphor Screens (GE Healthcare; BAS IP MS 2025 E, cat no. 28-9564-75) and scanned (GE Healthcare; Typhoon FLA 9500 IP, cat no. 29-1885-90).

#### Immunoprecipitation from Mouse Testes

Mouse MILI, MIWI2, MVH and MIWI antibodies were incubated with approximately 15 μL protein G-Sepharose beads (GE Healthcare, cat. No. 17-0618-01) overnight at 4°C and followed by washing (10 mM Tris-HCl pH 8.0, 150 mM NaCl, 0.05% NP-40) to remove the unbound antibody. The mouse testes were homogenized in a glass tissue homogenizer by douncing in lysis buffer [50 mM Tris-HCl pH 7.5, 150 mM NaCl, 5 mM MgCl_2_, 1 mM DTT, 0.5% sodium deoxycholate (Sigma, cat. No. 30968), 1% Triton X-100, 10% glycerol, 2 mM Ribonucleoside vanadyl complexes (Sigma, cat. no. R3380), protease inhibitor cocktail (Roche)] and spun down for 15 min at 4°C. 20-40 μl of clear lysate were eventually used for western-blot analysis. For immunoprecipitation, the beads were incubated with cleared testes lysate for 3 h and washed five times (10 mM Tris-HCl pH 8.0, 150 mM NaCl, 0.05% NP-40, 5% glycerol). The immunoprecipitated complex was further subjected to mass spectrometry or small RNA libraries preparation. Mass spectrometry of isolated MVH complexes was performed as described previously (Xiol et al., 2014).

Immunoprecipitations from P0 testes were carried out using lysates prepared with one pair of testes. The tissue was lysted in 1 ml of lysis buffer using tissue homogenizer and the lysate was spun down for 15 m in at 4°C. Supernatant was collected and used for isolation of MIWI2 complexes by incubation with antibody-bound beads. Subsequently, the same remaining supernatant after the MIWI2 isolation was used for purification of MILI complexes.

#### Small RNA Libraries

RNAs present in endogenous MILI, MIWI2, MIWI and MVH complexes were isolated. Briefly, immunoprecipitations were treated with Proteinase K in 300 μl reaction at 42°C for 15 min (10 mM Tris-HCl pH 7.5, 5 mM EDTA, 0.5% SDS). RNAs present in the sample were purified by phenol-chloroform extraction and precipitation with ethanol. Approximately 10-20% of the sample was labelled at the 5′ end with polynucleotide kinase (PNK, ThermoFisher Scientific) and [γ-^32^P] ATP (3000 Ci/mmol, Perkin Elmer), and resolved by 15% urea-PAGE for quality check.

We used 6 μl of the immunoprecipitated RNA for library preparation. Libraries were prepared (barcoded at 3′ end) using NEBNext® Multiplex Small RNA Library Prep Set for Illumina® (NEB Catalogue No. E7300) following manufacturer instructions. The synthesized cDNA libraries were resolved on 3% high-resolution MethaPhor agarose (Lonza, cat. No. 50180) gels in TAE buffer (Figure 1B). Fragments in the size-range of ∼160 bp (short libraries) and ∼200-300 bp (long libraries) were gel-extracted with the use of MinElute Gel Extraction Kit (Qiagen, cat No. 28604). Multiple libraries with different barcodes (at 3′end) were mixed in equimolar ratios and sequenced with the Illumina HiSeq 2000 platform (EMBL GeneCore facility, Heidelberg). The maximum sequencing length was 50 or 51 nt. Our library preparation strategy identifies the 5′ end of these sequences, irrespective of how long such sequences might be. All the generated datasets are listed in Table S1.

### Quantification and Statistical Analysis

#### Small RNA Data Analysis

Reads were sorted into individual libraries based on the barcodes, the 3′ adapter sequences were removed and mapped to the mouse genome (mm9). The software used for processing the data (genomic coordinates etc) from the raw data files are in-house tools developed by the Sachidanandam lab (Olson et al., 2008). Only reads perfectly matching the genome were kept for further analysis.

##### Analysis of Libraries from Adult Mvh Animals

After 3′ adapter sequence removal, the read length profile of short and long libraries was similar (data not shown). Only short libraries were used for further analysis. The read lengths of MILI-, MIWI- and MVH-associated sequences were plotted (Figures 4G and S5A). Whereas MILI piRNAs were preferentially 26-27 nt long, the MIWI piRNAs were mostly of 29-30 nt length (Figure 4G). In addition to the reads corresponding to the sizes of both MILI- and MIWI-bound piRNAs, the MVH libraries also contained reads of 51 nt, which was the maximum sequencing length (Figure 4K). We conclude that these reads are originating from longer RNA species present in MVH complexes (long reads).

To analyse the origin of MVH piRNAs, we annotated the MVH-associated 20-35 nt reads (Figure 4H). Most of these MVH piRNAs come from non-annotated (intergenic) regions with majority of them arising from top 50 pachytene piRNA clusters identified earlier (Reuter et al., 2011). The mm9 coordinates of the top 50 pachytene clusters used are given in Table S2. Based on the size distribution of MVH piRNAs we concluded that MVH preferentially associates with MIWI piRNAs (Figure 4G). To precisely define this, we grouped the piRNAs sharing their 5′ ends, and sorted them based on their preferential association with MILI, MIWI and MVH complexes using the following criteria. MILI-specific: at least 0.25 rpm in MILI (*Mvh*^*+*/*−*^) and at least 3x more abundant in MILI (*Mvh*^*+*/*−*^) than in MIWI (*Mvh*^*+*/*−*^). MIWI-specific: at least 0.25 rpm in MIWI (*Mvh*^*+*/*−*^) and at least 3x more abundant in MIWI (*Mvh*^*+*/*−*^) than in MILI (*Mvh*^*+*/*−*^). MILI and MIWI specific: at least 0.25 rpm in both MILI (*Mvh*^*+*/*−*^) and MIWI (*Mvh*^*+*/*−*^) with the difference less than 3x in between the libraries. MVH specific: at least 0.25 rpm in MVH (*Mvh*^*+*/*−*^) and not present in any of above categories. Using these criteria, we demonstrated that most of MVH piRNAs are those associating also with MILI and/or MIWI (Figure 4I).

We then compared the distribution of piRNAs and the long reads (51-mers) between the libraries. First, for the individual libraries we identified the genomic positions where the piRNAs or long reads start and applied a 0.25 rpm threshold to consider the 5′ end positions. We then calculated the percentage of the positions which were shared in between the individual libraries and the percentage of the piRNAs which originate at these positions (Figure S5D). We found that most of the piRNAs (MILI-, MIWI- and MVH-) originate from the same set of genomic sites. However, these comparisons did not reflect the abundance of piRNAs. Therefore, we calculated the Pearson correlation coefficient (PCC) for the abundance of the piRNAs sharing the 5′ end, and the PCC ^∗^100 was plotted (Figure 4J). Very high correlation was found when comparing the piRNA distribution between wild-type and *Mvh*^*+*/*KI*^, clearly demonstrating that the piRNA biogenesis is not affected in the mutant. The distribution of 5′ ends of MVH piRNAs is much better correlated with that of MIWI piRNAs than MILI piRNAs. Only low correlation was found between MVH piRNAs and MVH long RNAs.

To check whether the pachytene piRNAs might trigger slicing of target transcripts and lead to production of secondary piRNAs, we searched for the ping-pong signature (9 nt distance between the 5′ends) among the piRNAs targeting the transcripts and the piRNAs produced from the transcripts. The product of the piRNA counts was used to calculate the score for the 5′ end distance Δ: score(Δ)=ΣM(i)^∗^N(i+Δ), where M(i) is the count of produced piRNAs (in rpm) with 5′ end on the plus strand at a particular position i, and N(i+Δ) is the count of piRNAs which have their 5′ end position at minus strand at i + Δ. The distance equal to 0 refers to a situation where piRNAs share the 5′ end nucleotide and the distance 9 corresponds to 10nt overlap of piRNA 5′ ends. First, we analysed the 5′ end distances of targeting and produced reads by mapping the reads to L1_MM transposon consensus sequence (3 mismatches were allowed). The ping-pong signature was observed between MILI and MIWI piRNAs in both the wild-type and *Mvh*^*+*/*KI*^ mutant indicating the cleavage of L1 transcripts and production of secondary piRNAs (Figure S5F). It was also present between MILI (and MIWI) piRNAs and MVH long RNAs of the wild-type mouse demonstrating that MVH associates with the 5′ cleavage fragments resulting from slicer activity of MILI and MIWI. To find out whether the pachytene piRNAs might guide also the cleavage of genic transcripts (UCSC/mm9), we analyzed the ping-pong signatures between the piRNAs targeting the genic transcripts (3 mismatches allowed, with none of them at positions 2-10 of the piRNA) and the transcript-produced reads (no mismatch allowed). 500 transcripts with most targeted piRNAs were taken into account. We detected the ping-pong signature in between MILI and MIWI piRNAs and also in between the piRNAs and long RNAs associated with MVH (both wild-type and mutant) (Figures 4L and S5E).

##### Analysis of Libraries from P0 Mvh Animals

The read lengths of MILI- (Figure 3A) and MIWI2-associated (Figure S3A) reads from short libraries were plotted. Beside the piRNAs, the libraries also contained a population of contaminating miRNAs (peak at 22 nt). The libraries were normalized to the population of miRNAs (i.e. to get the normalized counts the read counts were divided by the count of 23 nt reads). MIWI2 piRNAs were absent in *Mvh*^−/KI^ and therefore only comparison between *Mvh*^+/−^ and *Mvh*^+/KI^ was possible, which showed no differences in length distribution, sense-antisense bias and annotation (Figures S3A–S3C). To analyse the MILI-associated piRNAs we filtered the reads of 24-30 nt size-range. Comparison of “sense piRNAs” (originating from annotated transcripts) and “antisense piRNAs” (targeting the transcripts), showed the overall decrease of “antisense” piRNAs in mutant *Mvh*^−/KI^ (Figure 3B). Annotation of piRNAs then showed that most of the piRNA classes are depleted in *Mvh*^−/KI^, with the antisense piRNAs being affected more than the sense piRNAs (Figure 3C). The depletion was accompanied by the increased proportion of piRNAs originating from genic transcripts (gene exon sense category).

We then compared the fractions of antisense piRNAs for top 20 individual repeat classes having most antisense piRNAs (the piRNAs mapping to tRNAs, rRNAs, snRNAs, scRNAs and srpRNAs were excluded). The depletion was apparent for most of the repeat classes (Figure 3D). The individual repeat comparison then demonstrated that IAPEY antisense piRNAs are strongly affected (the read counts plotted were normalized to miRNA levels; Figure 3E). The dramatic lack of piRNAs in the mutant was apparent also when mapping the piRNAs to the IAPEY consensus sequence (Figure S3F). Three mismatches were allowed and coverage of normalized counts was plotted – sense mapping as positive values and antisense as negative values. The 5′ end distances were calculated between the piRNAs targeting and originating from L1 and IAPEY consensus sequence, the score was calculated (see above) and the fraction of piRNA pairs having specific distance was plotted. Whereas L1 ping-pong signature (9nt peak) is weaker in the mutant *Mvh*^−/KI^, the ping-pong signature of IAPEY is completely gone (Figure 3G). Similar comparisons for repeat sense piRNAs showed the overall enrichment of L1 and Satellite piRNAs in the mutant *Mvh*^−/KI^ (Figures S3D and S3E). The enrichment was observed also when mapping the piRNAs to the L1 consensus (Figure S3F).

The observed overall increase of gene exon sense piRNAs led us to check the piRNA production of individual genes. Comparison of the normalized counts of produced piRNAs for individual genes demonstrated the overall increase of piRNAs (∼5 times) originating from most of the genes. SmoothScatter function from “graphics” R package was used to present the density representation of a scatterplot (Figure 3F).

To investigate whether we can identify the intermediates (16-mers and 50-mers – see below) generated by piRNA guided cleavage in the libraries, we compared the 5′-to-5′ distances or 3′-to-5′ distances between the L1 sense piRNAs and antisense mapped reads of different length. The score was calculated (see above) and the fraction of read pairs having specific distance was plotted (Figure S3H). The ping-pong signature between the sense and antisense piRNAs was apparent in all *Mvh* genotypes. Interestingly, in *Mvh*^−/KI^ we could identify the 16-mers and 50-mers whose 5′ or 3′ end, respectively is generated by sense piRNA guided cleavage.

##### Analysis P0 Mvh; Rosa26-pi Libraries

Short and long libraries were prepared from MILI-associated RNAs (Figure 1B). Reads were sorted into individual libraries based on the barcodes and the 3′ adapter sequences were clipped from the reads using cutadapt (DOI:http://dx.doi.org/10.14806/ej.17.1.200). Reads of at least 15 nucleotides were then aligned to the reporter sequence using bowtie (Langmead et al., 2009) allowing no mismatches. The reporter sequence consisted of the following elements: DsRed2 reporter-loxP-35 piRNA binding sites in a LacZ background-loxP-SV40 polyA signal.

Short libraries contained mainly piRNAs (peak at 26-27nt) and sequences of exactly 16 nt (16-mer). Long libraries additionally contained the reads of maximum sequencing length 51 nt (51-mer) which represent the 5′ portion of longer RNA species bound by MILI (Figures 1C and S1A).

The short libraries were used to compare the amount of reporter-derived piRNAs (24 nt-30 nt reads) and 16-mers between the wild-type and the *Mvh* mutant (Figures 3H and 3I). Read counts were normalized to library sizes and reads per million (rpm) were plotted. The long libraries were used to compare the 51-mers (Figures 3H and 3I). The reporter-produced piRNAs are drastically depleted in the *Mvh*^−/KI^ and *Mvh*^−/−^ mutants. The 16-mer and 51-mer also display slight reduction in the mutants, however this is not comparable to the decrease in piRNAs. To investigate the distribution of the piRNAs, we mapped the 5′ and 3′ ends of piRNAs, 3′ ends of 16-mers and 5′ ends of 51-mers along the reporter and calculated their distance from the closest site targeted by MILI piRNA (i.e. 5′ end of targeting piRNA). Therefore, the distance equal to 0 refers to position immediately downstream from 5′ end of targeting piRNA, the distance -10 refers to 10 nucleotide overlap with targeting piRNA (it corresponds to the 5′ fragment generated by MILI slicing). The counts of reads at specific distance were aggregated from all 35 MILI-targeted sites and their downstream sequences and plotted as rpm (Figures 1D, 1E, and S1B). Both secondary and inchworm (trail) piRNAs were detected and the 51-mers and 16-mers were identified as fragments generated by MILI slicing. We then plotted the individual sequenced reads of *Mvh*^+/−^, which were found in the vicinity of 2^nd^ target site (Figure 1F). Only the reads sequenced at least 6 times were shown with the color reflecting their abundance.

##### Analysis of Libraries from P0 Tdrd9 Animals

Only short libraries were prepared. The read lengths of MILI- and MIWI2-associated reads were plotted as a percentage of the library reads (Figure S8A). The piRNAs of 24-30nt were filtered and their amount compared between the samples. The piRNAs were divided into groups based on the nucleotide at their 1^st^ and 10^th^ position. No obvious difference was found between the wild-type (*Tdrd9*^+/KI^) and the mutants (*Tdrd9*^KI/KI^ and *Tdrd9*^−/−^). The piRNA genome annotation showed the enrichment of repeat piRNAs associated with MILI in the mutants (*Tdrd9*^KI/KI^ and *Tdrd9*^−/−^) (Figure S8B). Then we compared the amount of piRNAs (as percentage of the libraries) associated with individual repeats (Figures 5K and S8C). Only repeats associated with at least 10 rpm in one of the compared samples were plotted. We observed enrichment of L1 and LTR piRNAs associated with MILI in the mutants, whereas the SINE piRNAs associated with MIWI2 were depleted (Figure S8D). The lack of SINE-targeting MIWI2 piRNAs was also apparent when mapping the piRNAs to B1Mus1.SINE transposon consensus (Figure 5L). Three mismatches were allowed and the 5′ end piRNA distribution was plotted: the sense mapping as positive values and antisense as negative values. The overall counts of genome mapped piRNAs annotated to SINE, LINE and LTR elements were plotted in Figure S8D.

### Data and Software Availability

Deep sequencing data generated in this study are deposited with Gene Expression Omnibus under the accession number GEO: GSE95580. Mouse mutants generated in this study are deposited with the European Mouse Mutant Archive (EMMA) under the accession numbers: *Tdrd9*^*E257Q*^ (EM:08489) and *Mvh*^*E446Q*^ (EM:09169). Both mouse lines can be crossed with Cre mice to obtain the respective knockout lines.

## Author Contributions

Z.Y. initiated the *Mvh* study by creating mouse mutants. J.M.W. performed all analyses with help from Z.Y. All computational analyses were done by D.H. with help from R.S. Immunoprecipitations and deep sequencing libraries were done by R.R.P. Creation and analysis of *Tdrd9* mutants were by P.S. Coordination and manuscript preparation were by R.R.P. and R.S.P., with input from others.

## Supplemental Information

Document S1. Figures S1–S8 and Tables S1 and S2

Document S2. Article plus Supplemental Information
