# Supplementary material for: Distinct Roles of RNA Helicases MVH and TDRD9 in PIWI Slicing-Triggered Mammalian piRNA Biogenesis and Function
Source: Dev Cell. 2017 Jun 19;41(6):623–637.e9. doi: 10.1016/j.devcel.2017.05.021 (PMC5481186; doi:10.1016/j.devcel.2017.05.021)
Supplement: Document S1. Figures S1–S8 and Tables S1 and S2 [file mmc1.pdf]

Developmental Cell, Volume *41*

Supplemental Information

Distinct Roles of RNA Helicases MVH and TDRD9 in PIWI Slicing-Triggered Mammalian piRNA Biogenesis and Function

Joanna M. Wenda, David Homolka, Zhaolin Yang, Pietro Spinelli, Ravi Sachidanandam, Radha Raman Pandey, and Ramesh S. Pillai

SUPPLEMENTAL FIGURE LEGENDS

Figure S1. MILI slicer products are present in *Mvh*$^{-/KI}$ and *Mvh*$^{-/-}$ mutants, but these fail to mature into piRNAs. Related to Figure 1 and 3.

(A) Mice carrying the artificial piRNA precursor (*Rosa26-pi*) in various *Mvh* genetic backgrounds were used for isolation of MILI complexes and preparation of deep sequencing libraries. Testes from newborn pups (P0) were used. Libraries containing short and long reads were sequenced separately (see STAR Methods). Length (in nucleotides, nt) distribution of the reporter-derived reads is compared between the various mutants. The piRNAs are almost completely absent in *Mvh*$^{-/KI}$ and *Mvh*$^{-/-}$ animals. (B) Mapping of the 5′ and 3′ ends of the reporter-derived reads relative to the 5′ end (nucleotide position −1) of the targeting MILI piRNA. Although there is an overall drastic reduction of piRNAs in the *Mvh*$^{-/KI}$ and *Mvh*$^{-/-}$ mutants, the residual secondary and inchworm (or trail) piRNAs reveal similar origins.

Figure S2. Creation of the *Mvh* catalytic-dead knock-in mouse. Related to Figure 2.

(A) Protein sequence alignment of Vasa orthologues from insects to human. The residues forming the ATP binding (GKT) and ATP hydrolysis (DEAD) motifs are indicated with arrowheads. Secondary structure features as present in the crystal structure of DmVasa is shown above the sequences (PDB: 2DB3). (B) Gene targeting in mouse ES cells to introduce a point mutation (E446Q) in exon 16 (indicated with red asterisk) of the *Mvh* gene locus. The targeting construct also brought in a neomycin selection cassette and *loxP* sites flanking the exon 16, for creation of the knock-out allele. The selection cassette was removed in all the experimental mice. Position of genotyping primers is shown (green arrows). (C) Southern blotting to confirm gene targeting in mouse ES cells. One of these targeted cell clones was used for mouse generation. (D) Ethidium bromide-stained gel showing the genomic PCR fragments obtained during a routine genotyping strategy using mouse tail genomic DNA. Some of the animals had both the *Mvh* allele and the *Rosa26-pi* reporter. (E) DNA sequencing tracks from one of the genotyping PCR products to show the presence of the E446Q mutation. Note that in the *Mvh*$^{+/KI}$ genotype, contribution from both the wildtype and mutant alleles can be seen.

Figure S3. Analysis of MIWI2- and MILI-bound piRNAs from different *Mvh* genotypes. Related to Figure 3.

(A) Length distributions of RNAs co-immunoprecipitated with MIWI2 from *Mvh*$^{+/-}$ and *Mvh*$^{+/KI}$ P0 mouse testes. Majority of the reads refer to piRNAs (29-30 nt peak). A contaminating population of miRNAs is marked with an arrowhead (22 nt peak). (B) MIWI2-associated piRNAs (24-30 nt) were divided into “sense”, originating from annotated transcripts, and “antisense”, targeting the transcripts. Their counts were normalized to miRNA levels and compared. (C) Genome annotations of MIWI2 piRNA reveal similar distributions for *Mvh*$^{+/-}$ and *Mvh*$^{+/KI}$ genotypes. (D) MILI-associated piRNAs were isolated from P0 testes and analysed by deep sequencing. Counts of MILI piRNAs produced from individual repeat classes are shown. Whereas many repeat classes display reduced piRNA levels in the *Mvh*$^{-/KI}$ mutant, levels of L1 and Satellite piRNAs are increased. (E) Normalized counts of sense-oriented piRNAs are compared for individual repeats. (F) The graphs show the distribution of piRNAs mapped along L1 and IAPEYI consensus sequences. Coverage of targeting piRNAs is shown as negative values and coverage of sense-oriented piRNAs is displayed as positive values. While the production of sense-oriented L1 piRNAs is increased in the *Mvh*$^{-/KI}$ mutant, likely due to transposon de-repression and increased availability of such substrates, the piRNAs targeting the IAPEYI sequence are almost absent. (G) Length distributions of RNAs immunoprecipitated with MILI from P0 testes and mapping to L1 transposon consensus sequence in antisense orientation. Majority of the reads refer to piRNAs (25-30 nt), however the full-length reads of 50

nt (50-mers) and short 16 nt reads (16-mers) are also present, and enriched in *Mvh*$^{-/KI}$. (H) To study whether the detected L1 antisense reads (piRNAs,16-mers and 50-mers) are generated by piRNA guided cleavage, we compared the distances between the 5′ end of targeting sense piRNAs and the 5′ or 3′ ends of antisense reads. The cleavage takes place in 9 nt distance from the 5′ end of targeting piRNA and creates the 5′ end of antisense secondary piRNA in all *Mvh* genotypes. The cleavage also defines the 5′ and 3′end of the 50- and 16-mer, respectively, which are exclusively observed in *Mvh*$^{-/KI}$. The 16- and 50-mers therefore represent the intermediate cleavage products of endogenous slicer targets.

Figure S4. Biogenesis of piRNAs is unaffected in the dominant-negative *Mvh*$^{+/KI}$ mutant. Related to Figure 4.

(A) Immunofluorescence detection of γ-H2AX in adult *Mvh*$^{+/KI}$ testes. Arrows point to the XY body in spermatocytes. (B) TUNEL assay to detect double-stranded DNA breaks (green signal) in adult mouse testes of indicated genotypes. Note the increased signal in the *Mvh* mutants that display arrested spermatogenesis, and consequent infertility. (C) Western analysis of MVH in total testes lysates from adult animals of indicated genotypes. ACTIN is used as loading control. Note that the reduced MVH levels in the *Mvh*$^{-/KI}$ mutant is due to an early arrest of spermatogenesis and loss of late-stage germ cells due to apoptosis (see below). (D) Subcellular localization of MVH, MILI and MIWI to a singular perinuclear cytoplasmic granule called the chromatoid body in purified round spermatids. These cells were isolated from adult testes of indicated genotypes. (E) Immunoprecipitation of MVH and PIWI proteins from adult mouse testes of indicated genotypes. The associated RNAs were revealed by 5′ end labelling. Note the presence of piRNAs in MVH complexes isolated from the *Mvh*$^{+/KI}$ mutant, but not seen in the control *Mvh*$^{+/-}$ testes. Association of piRNAs with MILI and MIWI seems not be affected. Sequence analysis also revealed unchanged composition (see Figure 4).

Figure S5. Analysis of slicer products associating with MVH in the *Mvh* dominant-negative mutant. Related to Figure 4.

(A) Length distributions of piRNAs immunoprecipitated with MILI and MIWI from adult mouse testes. (B) Relative proportion of individual annotation categories was compared between MVH-associated long reads (51-mers) and MVH-associated piRNAs. (C) The frequency of individual nucleotides at 1st and 10th position is shown for piRNAs bound by MILI, MIWI and MVH. There is a strong 1U-bias, a feature found in pachytene piRNAs. (D) The genomic coordinates of 5′ ends of piRNAs and MVH-associated long reads (51-mers) were identified and those generating at least 0.25 rpm were considered. The percentage of the shared 5′ end positions is shown for individual library comparisons (left panel). Percentage of the reads which originate from those positions in each library is shown (right panel). For example, ~70% of positions where MIWI$^{+/KI}$ piRNAs start also give rise to MILI$^{+/-}$ piRNAs and ~90% of all MIWI$^{+/KI}$ piRNAs start from these positions. (E) The plots show the 5′ end distance between the piRNAs targeting the genic transcripts and the transcript-derived long reads (51-mers) that are associated with MVH. (F) The plots show the 5′ end distances between the piRNAs targeting the L1_MM transposon consensus and originating piRNAs or the the transposon-derived long reads (51-mers) that are associated with MVH.

Figure S6. Creation of the *Tdrd9* catalytic-dead knock-in mouse. Related to Figure 5.

(A) Gene targeting strategy for introduction of point mutation E247Q (indicated with an asterisk) in the ATPase motif (DEVH→DQVH) of the exon 5 of the *Tdrd9* locus in mouse ES cells. Introduction of *loxP* sites also allow creation of the null allele by deletion of exons 3-5. (B) Southern blot analysis of genomic DNA from targeted ES cell clones to determine successful gene targeting. (C) Genomic PCR strategy to identify mutant alleles from mouse tail genomic DNA. Sequence tracks from such PCR fragments is shown to reveal the presence of the point mutation E247Q.

Figure S7. Analysis of spermatogenesis and transposon silencing in *Tdrd9* knock-in mice. Related to Figure 5.

(A) Hematoxylin and eosin staining of adult mouse testes from indicated *Tdrd9* knock-in genotypes. Notice the large seminiferous tubules in the *Tdrd9*$^{+/KI}$ control, while those in the *Tdrd9*$^{KI/KI}$ mutant are small and narrow due to arrested spermatogenesis. A zoom-in view of the control testes shows presence of elongate spermatids with condensed nuclei. (B) Staining of P18 (18-day old) mouse testes for γ-H2AX. The zoomed views show presence of the XY body in control *Tdrd9*$^{+/KI}$ testes, but not in the *Tdrd9*$^{KI/KI}$ mutant. Notice the increased general staining in the mutant germ cells, likely indicating increased DNA damage. (C) TUNEL assay revealing increased dsDNA breaks (green signal) in the *Tdrd9*$^{KI/KI}$ mutant, suggesting a loss of germ cells by apoptosis after spermatogenic arrest. (D) Immunofluorescence detection of L1ORF1p protein in the *Tdrd9*$^{KI/KI}$ mutant germ cells, but not in the control *Tdrd9*$^{+/KI}$ testes. Scale bars are indicated.

Figure S8. Lack of piRNA biogenesis defect in the *Tdrd9* catalytic-dead mutant. Related to Figure 5.

MILI and MIWI2 complexes were isolated from P0 animals of the indicated genotypes and examined by deep sequencing. (A) The read length profile of both MILI and MIWI2 piRNAs is similar between *Tdrd9*$^{+/KI}$, *Tdrd9*$^{KI/KI}$ and *Tdrd9*$^{-/-}$, as well as the proportion of primary (1U) and secondary (A10) piRNAs which were defined by the presence of specific nucleotide preferences. (B) Comparison of genome annotation for individual libraries. The loss of TDRD9 or presence of a catalytic-dead mutation in TDRD9 results in accumulation of repeat piRNAs in MILI. (C) Comparison of MIWI2-associated piRNAs (as percentage of the library reads) mapped to individual repeats. While LINE and LTR piRNA counts are increased in *Tdrd9* mutants, the levels of SINE piRNAs are decreased. (D) Comparison of the amount of piRNAs mapped to SINE, LTR and LINE elements. Whereas MIWI2-associated SINE piRNAs are depleted in *Tdrd9*$^{KI/KI}$ and *Tdrd9*$^{-/-}$ mice, MILI-associated LINE and LTR piRNAs are enriched. Thus, although piRNA levels fluctuate, most antisense piRNAs targeting active transposons are still present in the catalytic-dead *Tdrd9* mutant.

Supplemental Table 1. List of all deep-sequencing libraries created in this study. Related to STAR Methods and Figure 1.

Data is available from GEO under accession no. GSE95580.

Supplemental Table 2. List of the top 50 mouse pachytene piRNA clusters used for data analysis. Related to STAR Methods and Figure 4.

Genomic coordinates of the clusters based on the mm9 mouse assembly is indicated.

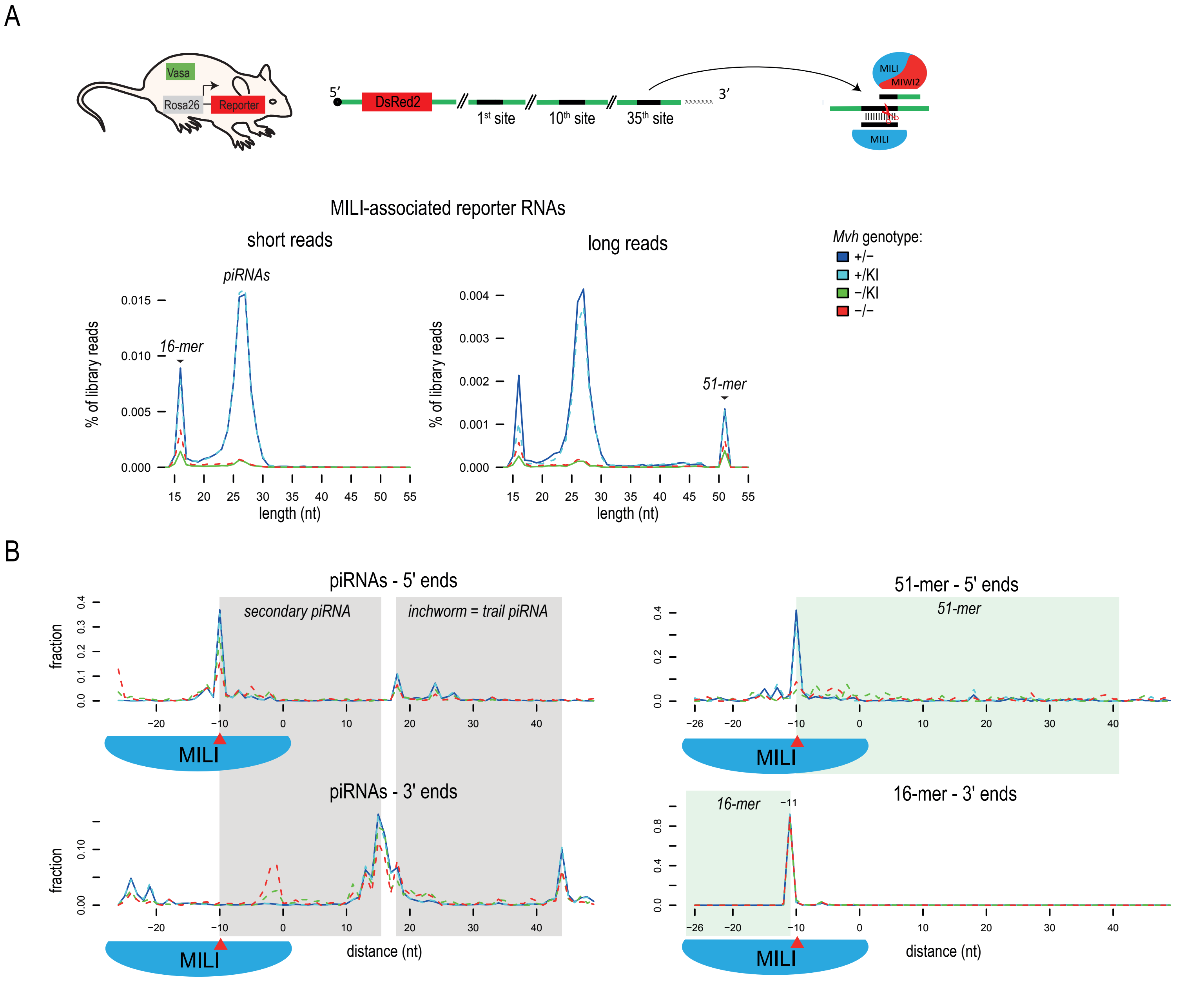
A
Vasa
Rosa26
Reporter
5′
DsRed2
1st site
10th site
35th site
3′
MILI
MIWI2
MILI
MILI-associated reporter RNAs
short reads
long reads
piRNAs
16-mer
51-mer
% of library reads
length (nt)
Mvh genotype:
+/−
+/KI
−/KI
−/−
B
piRNAs - 5' ends
secondary piRNA
inchworm = trail piRNA
51-mer - 5' ends
51-mer
piRNAs - 3' ends
16-mer - 3' ends
16-mer
fraction
distance (nt)
MILI

Figure- S1

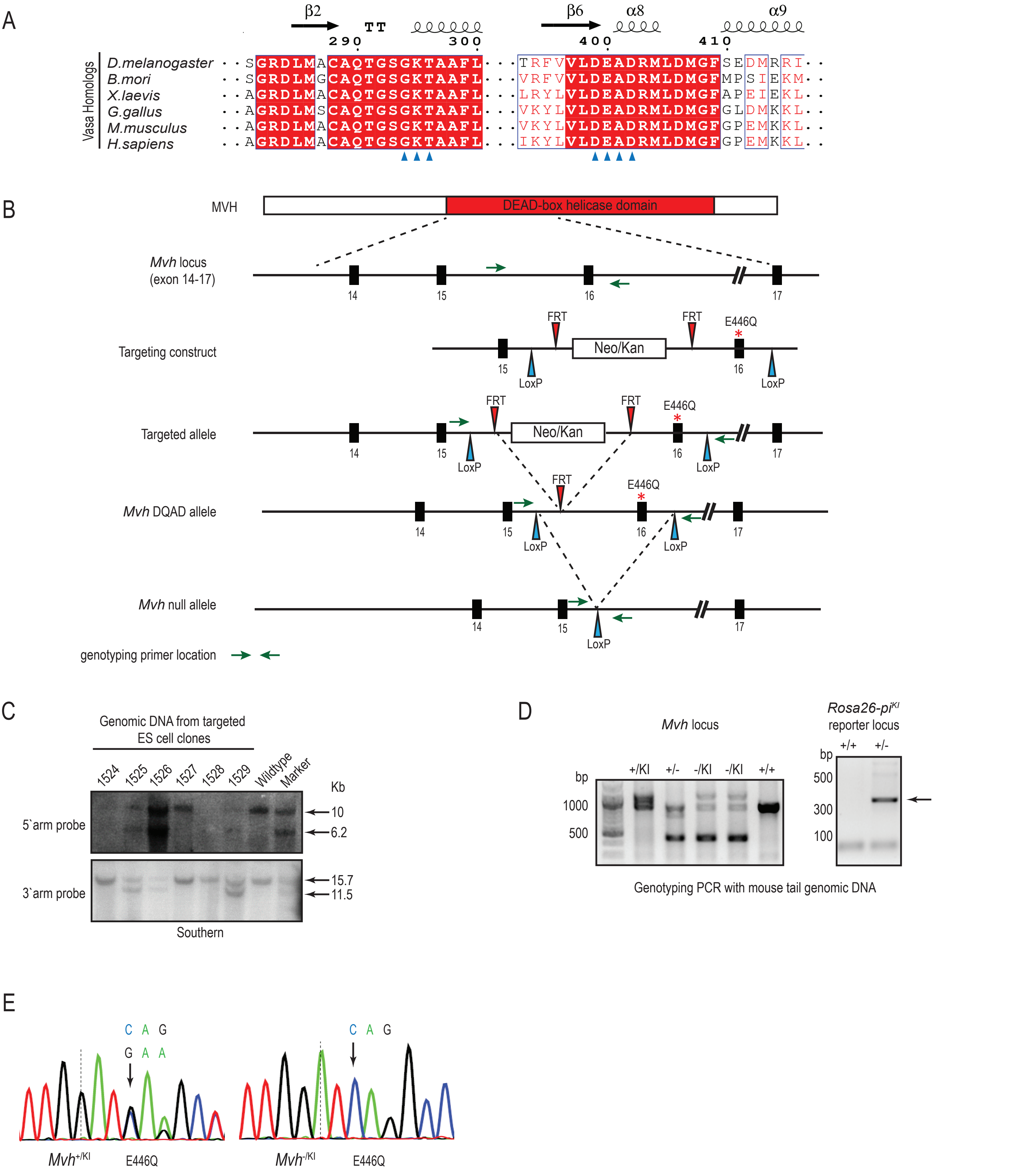
A
β2
TT
β6
α8
α9
290
300
400
410
Vasa Homologs
D.melanogaster
B.mori
X.laevis
G.gallus
M.musculus
H.sapiens
SGRDLMACAQTGSGKTAAFL
SGRDLMGCAQTGSGKTAAFL
AGRDLMACAQTGSGKTAAFL
AGRDLMSCAQTGSGKTAAFL
AGRDLMACAQTGSGKTAAFL
AGRDLMACAQTGSGKTAAFL
TRFVVLDEADRMLDMGFSEDMRRI
VRFVVLDEADRMLDMGFMPSIEKM
LRYLVLDEADRMLDMGFAPEIEKL
VKYLVLDEADRMLDMGFGLDMKKL
VKYLVLDEADRMLDMGFGPEMKKL
IKYLVLDEADRMLDMGFGPEMKKL
B
MVH
DEAD-box helicase domain
Mvh locus
(exon 14-17)
14
15
16
17
Targeting construct
FRT
Neo/Kan
E446Q
LoxP
Targeted allele
Mvh DQAD allele
Mvh null allele
genotyping primer location
C
Genomic DNA from targeted
ES cell clones
1524
1525
1526
1527
1528
1529
Wildtype
Marker
Kb
5`arm probe
3`arm probe
10
6.2
15.7
11.5
Southern
D
Mvh locus
Rosa26-pi^KI
reporter locus
bp
+/KI
+/-
-/KI
-/KI
+/+
1000
500
500
300
100
Genotyping PCR with mouse tail genomic DNA
E
C A G
G A A
Mvh+/KI
E446Q
Mvh-/KI
E446Q

Figure- S2

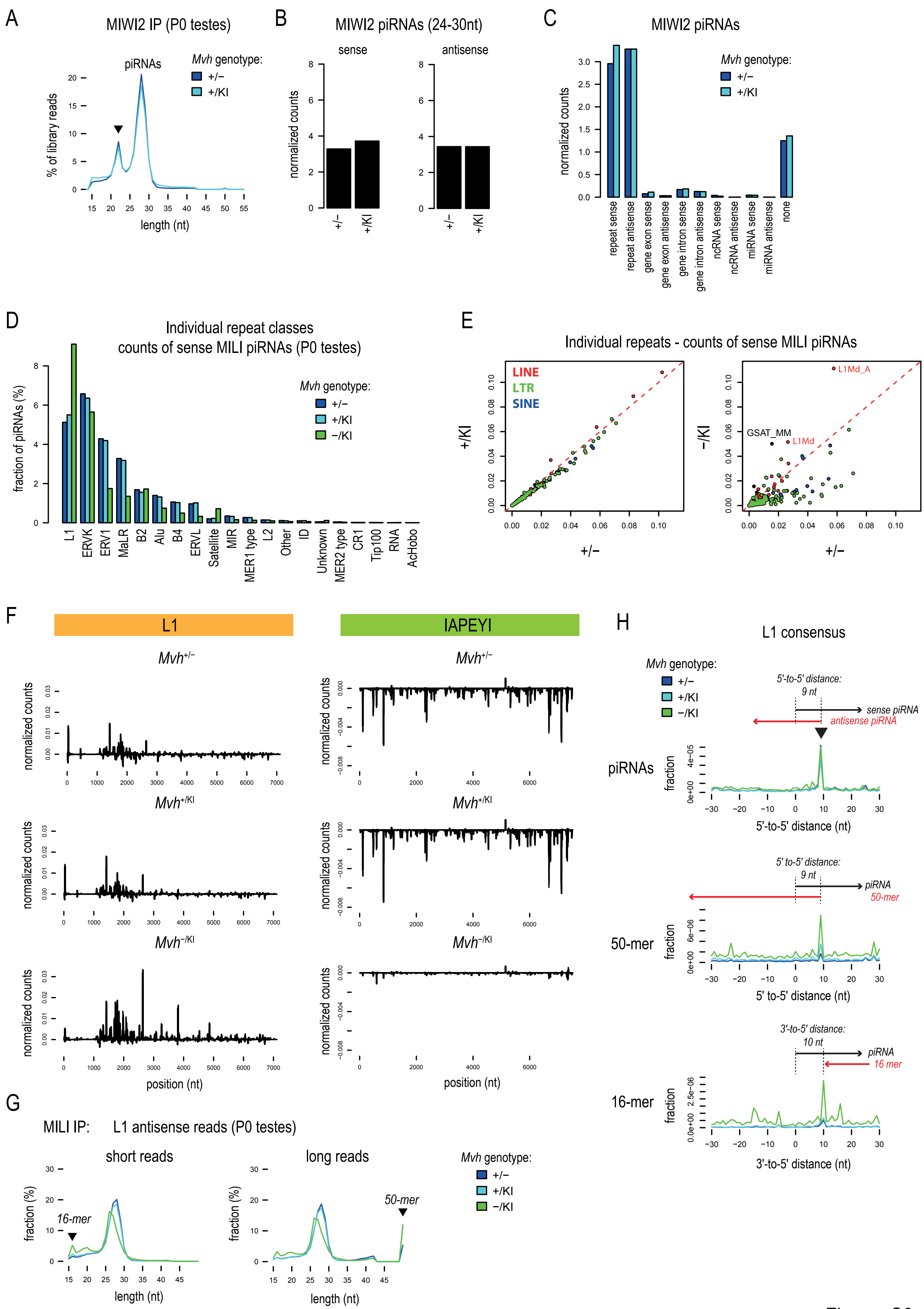

A
MIWI2 IP (P0 testes)
piRNAs
Mvh genotype:
+/−
+/KI
% of library reads
length (nt)
B
MIWI2 piRNAs (24-30nt)
sense
antisense
normalized counts
C
MIWI2 piRNAs
Mvh genotype:
+/−
+/KI
normalized counts
repeat sense
repeat antisense
gene exon sense
gene exon antisense
gene intron sense
gene intron antisense
ncRNA sense
ncRNA antisense
miRNA sense
miRNA antisense
none
D
Individual repeat classes
counts of sense MILI piRNAs (P0 testes)
Mvh genotype:
+/−
+/KI
−/KI
fraction of piRNAs (%)
L1
ERVK
ERV1
MaLR
B2
Alu
B4
ERVL
Satellite
MIR
MER1 type
L2
Other
ID
Unknown
MER2 type
CR1
Tip100
RNA
AcHobo
E
Individual repeats - counts of sense MILI piRNAs
LINE
LTR
SINE
+/KI
−/KI
+/−
L1Md_A
GSAT_MM
L1Md
F
L1
IAPEYI
Mvh+/−
Mvh+/KI
Mvh−/KI
normalized counts
position (nt)
G
MILI IP: L1 antisense reads (P0 testes)
short reads
long reads
16-mer
50-mer
fraction (%)
length (nt)
Mvh genotype:
+/−
+/KI
−/KI
H
L1 consensus
Mvh genotype:
+/−
+/KI
−/KI
5'-to-5' distance:
9 nt
sense piRNA
antisense piRNA
piRNAs
fraction
5'-to-5' distance (nt)
5' to-5' distance:
9 nt
piRNA
50-mer
5' to-5' distance (nt)
3'-to-5' distance:
10 nt
piRNA
16 mer
16-mer
3'-to-5' distance (nt)

Figure- S3

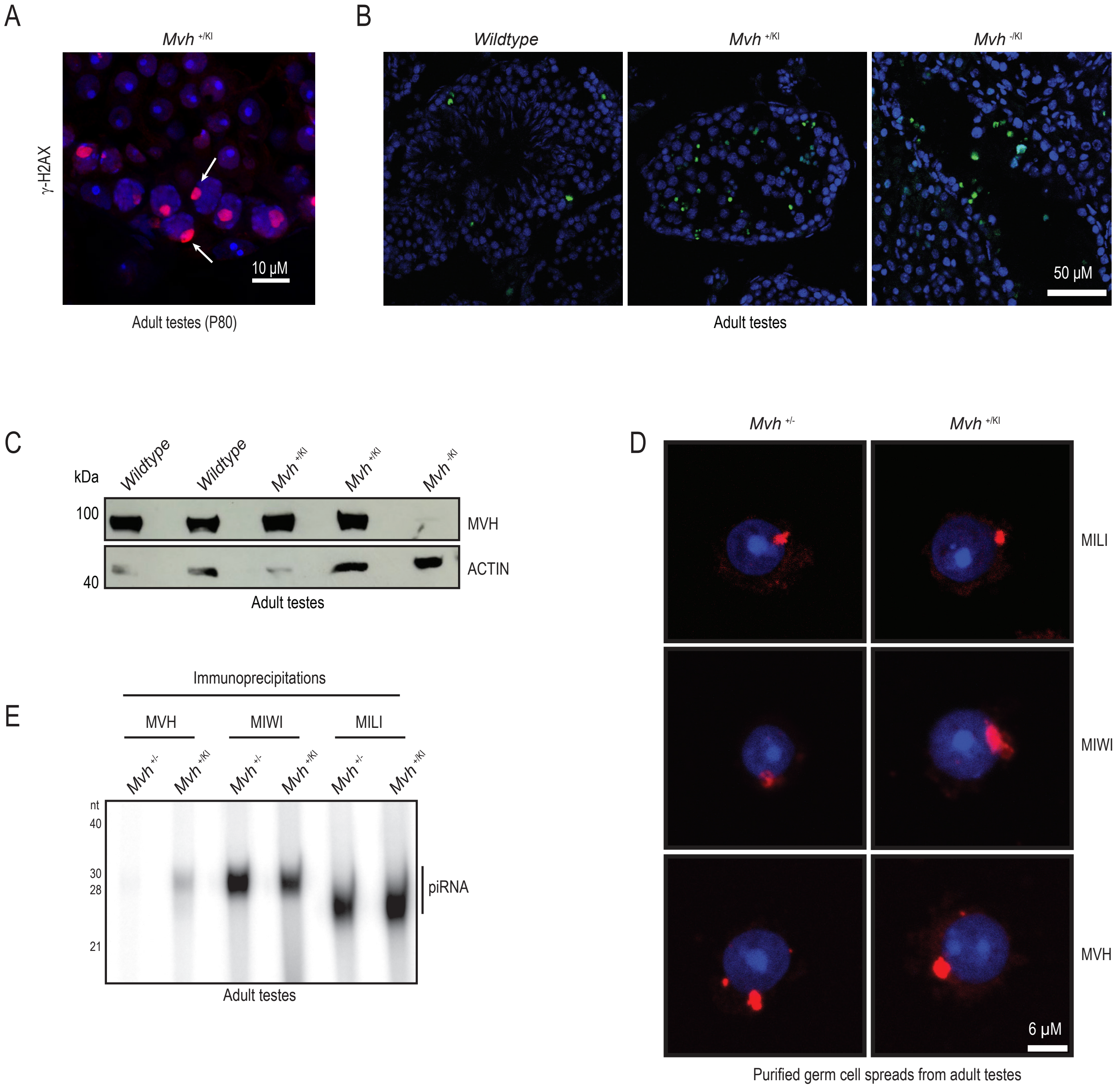
A
Mvh +/KI
γ-H2AX
10 μM
Adult testes (P80)
B
Wildtype
Mvh +/KI
Mvh -/KI
50 μM
Adult testes
C
kDa
Wildtype
Wildtype
Mvh +/KI
Mvh +/KI
Mvh -/KI
100
40
MVH
ACTIN
Adult testes
D
Mvh +/-
Mvh +/KI
MILI
MIWI
MVH
6 μM
Purified germ cell spreads from adult testes
E
Immunoprecipitations
MVH
MIWI
MILI
Mvh +/-
Mvh +/KI
Mvh +/-
Mvh +/KI
Mvh +/-
Mvh +/KI
nt
40
30
28
21
piRNA
Adult testes

Figure- S4

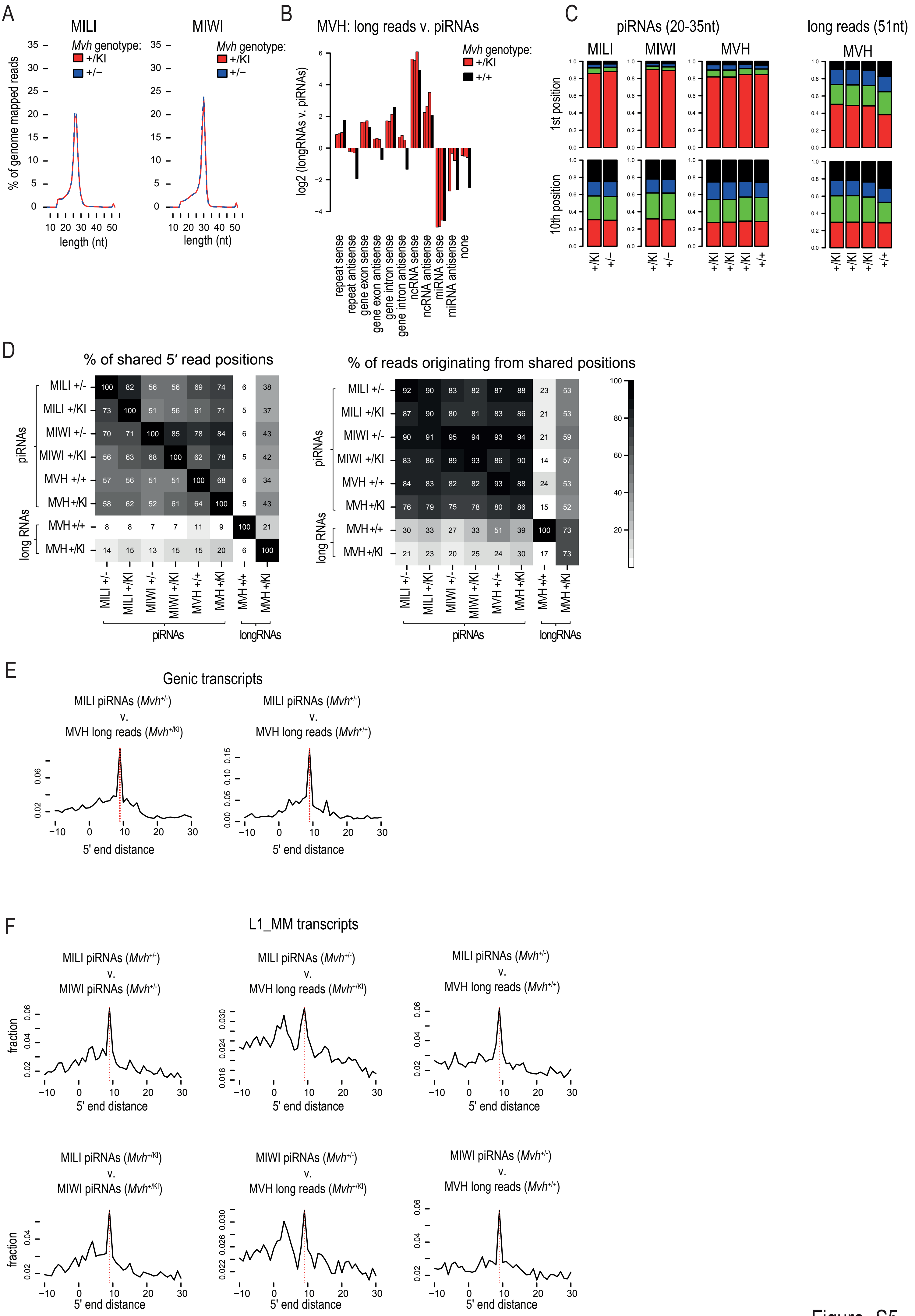
A
MILI
MIWI
Mvh genotype:
+/KI
+/−
% of genome mapped reads
length (nt)
B
MVH: long reads v. piRNAs
log2 (longRNAs v. piRNAs)
repeat sense
repeat antisense
gene exon sense
gene exon antisense
gene intron sense
gene intron antisense
ncRNA sense
ncRNA antisense
miRNA sense
miRNA antisense
none
+/+
C
piRNAs (20-35nt)
long reads (51nt)
MVH
1st position
10th position
D
% of shared 5′ read positions
MILI +/- 100 82 56 56 69 74 6 38
MILI +/KI 73 100 51 56 61 71 5 37
MIWI +/- 70 71 100 85 78 84 6 43
MIWI +/KI 56 63 68 100 62 78 5 42
MVH +/+ 57 56 51 51 100 68 6 34
MVH +/KI 58 62 52 61 64 100 5 43
MVH +/+ 8 8 7 7 11 9 100 21
MVH +/KI 14 15 13 15 15 20 6 100
% of reads originating from shared positions
MILI +/- 92 90 83 82 87 88 23 53
MILI +/KI 87 90 80 81 83 86 21 53
MIWI +/- 90 91 95 94 93 94 21 59
MIWI +/KI 83 86 89 93 86 90 14 57
MVH +/+ 84 83 82 82 93 88 24 53
MVH +/KI 76 79 75 78 80 86 15 52
MVH +/+ 30 33 27 33 51 39 100 73
MVH +/KI 21 23 20 25 24 30 17 73
piRNAs
long RNAs
longRNAs
E
Genic transcripts
MILI piRNAs (Mvh+/-) v. MVH long reads (Mvh+/KI)
MILI piRNAs (Mvh+/-) v. MVH long reads (Mvh+/+)
5' end distance
F
L1_MM transcripts
MILI piRNAs (Mvh+/-) v. MIWI piRNAs (Mvh+/-)
MILI piRNAs (Mvh+/-) v. MVH long reads (Mvh+/KI)
MILI piRNAs (Mvh+/-) v. MVH long reads (Mvh+/+)
MILI piRNAs (Mvh+/KI) v. MIWI piRNAs (Mvh+/KI)
MIWI piRNAs (Mvh+/-) v. MVH long reads (Mvh+/KI)
MIWI piRNAs (Mvh+/-) v. MVH long reads (Mvh+/+)
fraction
5' end distance

Figure- S5

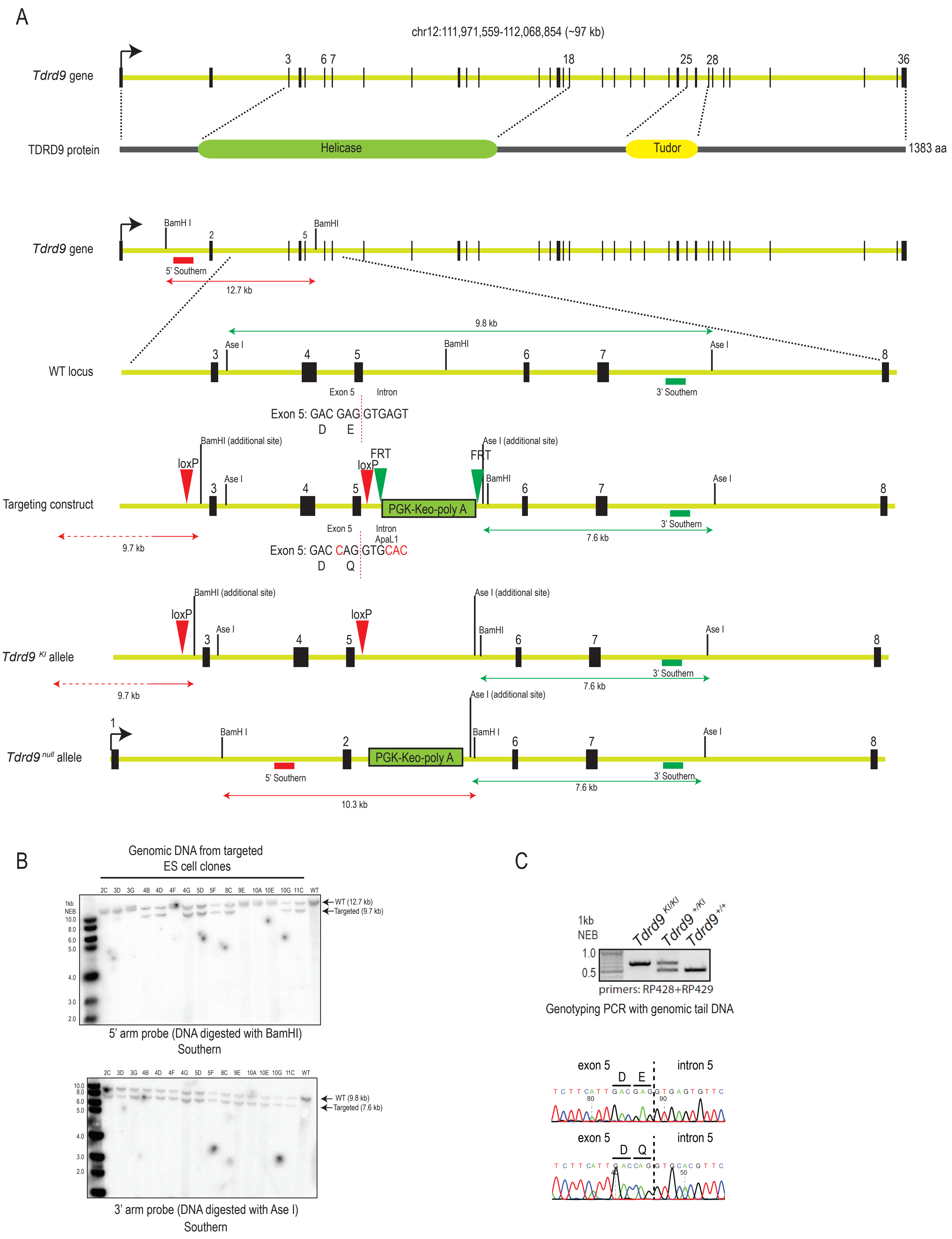
A
chr12:111,971,559-112,068,854 (~97 kb)
Tdrd9 gene
TDRD9 protein
Helicase
Tudor
1383 aa
BamH I
BamHI
5' Southern
12.7 kb
9.8 kb
WT locus
Ase I
3' Southern
Exon 5
Intron
Exon 5: GAC GAG GTGAGT
D E
Targeting construct
BamHI (additional site)
Ase I (additional site)
loxP
FRT
PGK-Keo-poly A
7.6 kb
9.7 kb
Intron
ApaL1
Exon 5: GAC CAG GTGCAC
D Q
Tdrd9 KI allele
Tdrd9 null allele
10.3 kb
B
Genomic DNA from targeted ES cell clones
2C 3D 3G 4B 4D 4F 4G 5D 5F 8C 9E 10A 10E 10G 11C WT
1kb NEB
WT (12.7 kb)
Targeted (9.7 kb)
5' arm probe (DNA digested with BamHI) Southern
WT (9.8 kb)
Targeted (7.6 kb)
3' arm probe (DNA digested with Ase I) Southern
C
1kb NEB
Tdrd9 KI/KI
Tdrd9 +/KI
Tdrd9 +/+
1.0
0.5
primers: RP428+RP429
Genotyping PCR with genomic tail DNA
exon 5
intron 5
D E
D Q

Figure-S6

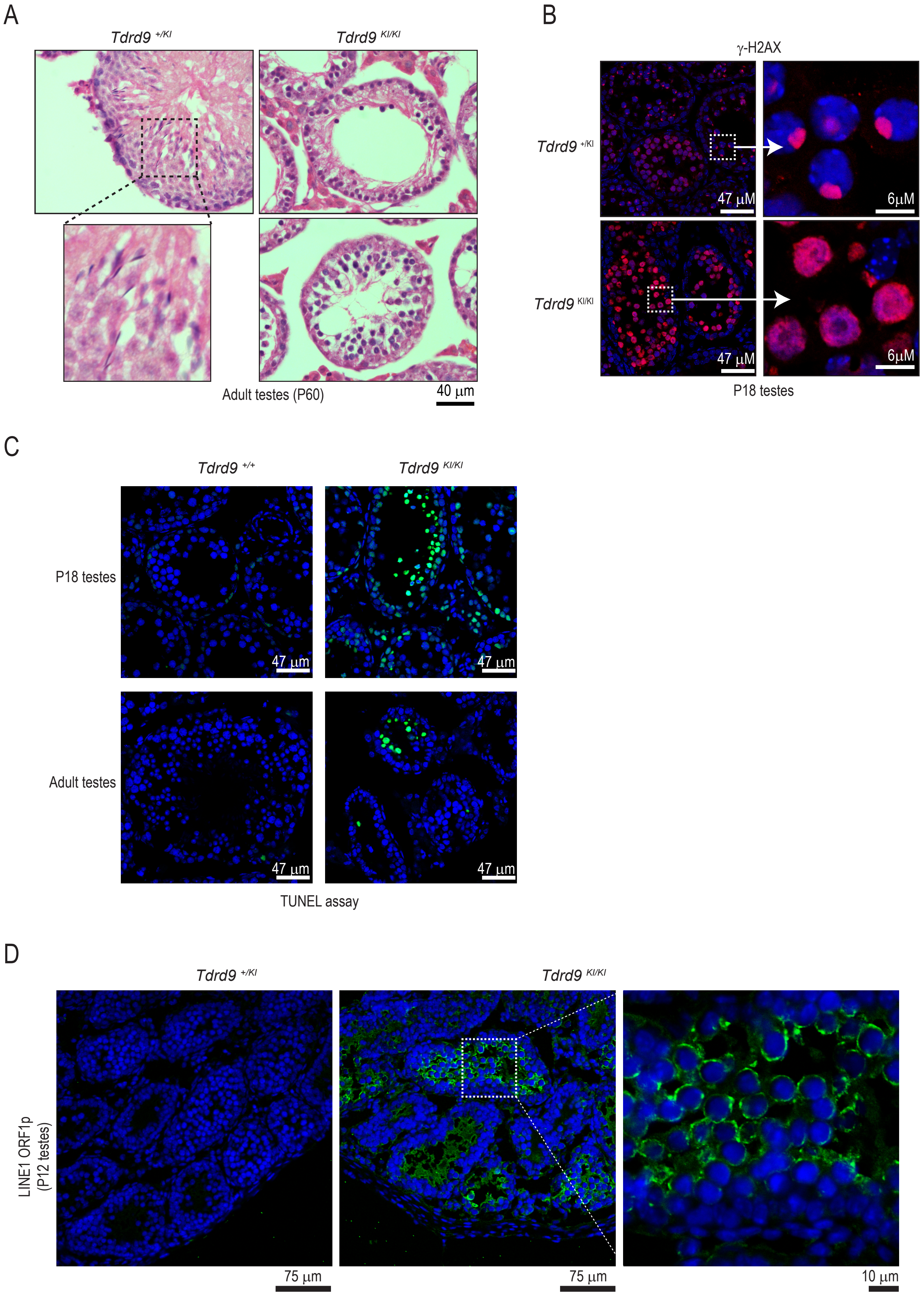
A
Tdrd9 +/KI
Tdrd9 KI/KI
Adult testes (P60)
40 µm
B
γ-H2AX
Tdrd9 +/KI
Tdrd9 KI/KI
47 µM
6µM
P18 testes
C
Tdrd9 +/+
Tdrd9 KI/KI
P18 testes
Adult testes
47 µm
TUNEL assay
D
Tdrd9 +/KI
Tdrd9 KI/KI
LINE1 ORF1p
(P12 testes)
75 µm
10 µm

Figure- S7

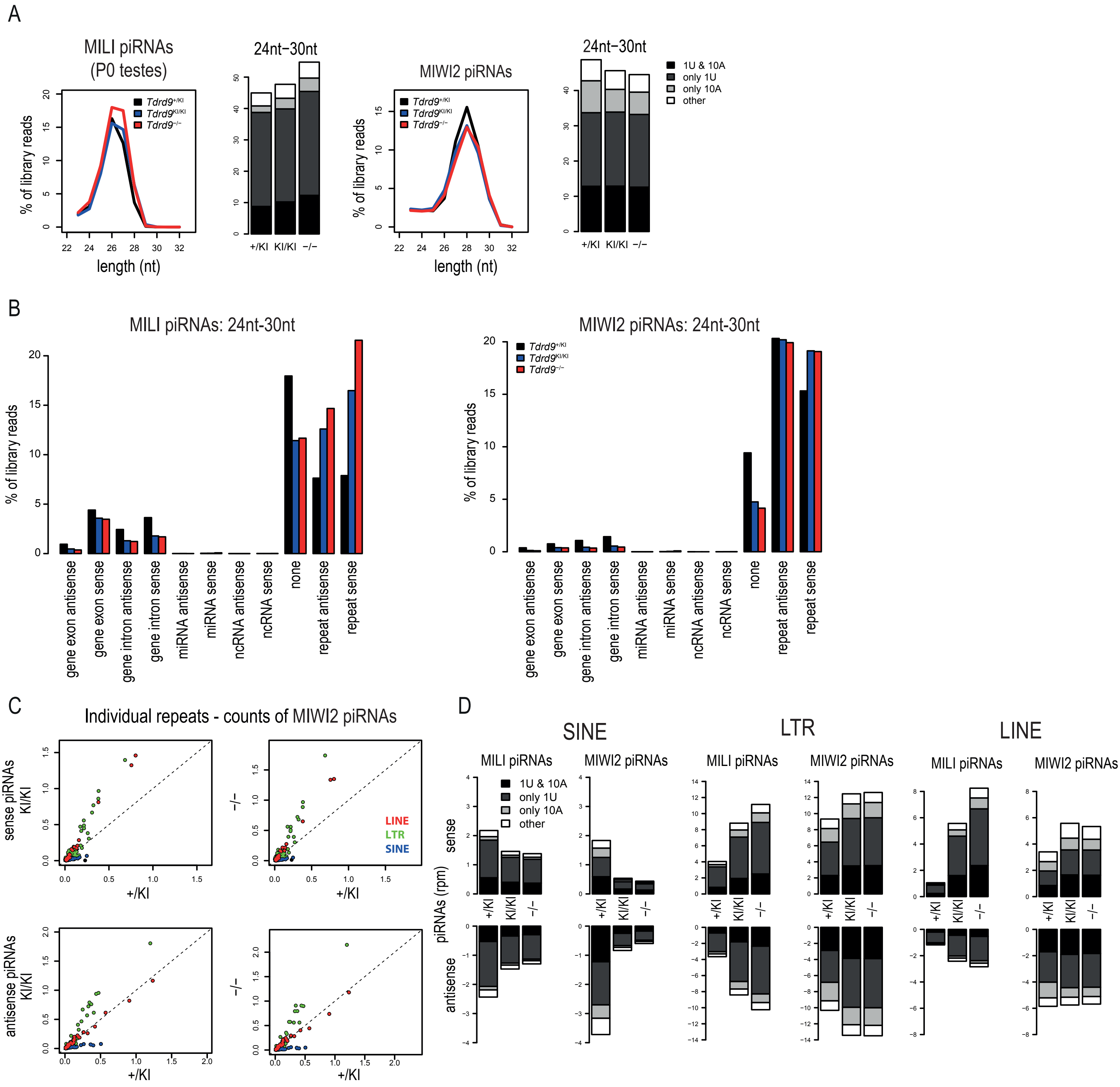
A
MILI piRNAs
(P0 testes)
Tdrd9+/KI
Tdrd9KI/KI
Tdrd9−/−
% of library reads
length (nt)
24nt−30nt
+/KI KI/KI −/−
MIWI2 piRNAs
24nt−30nt
1U & 10A
only 1U
only 10A
other
B
MILI piRNAs: 24nt-30nt
MIWI2 piRNAs: 24nt-30nt
% of library reads
gene exon antisense
gene exon sense
gene intron antisense
gene intron sense
miRNA antisense
miRNA sense
ncRNA antisense
ncRNA sense
none
repeat antisense
repeat sense
C
Individual repeats - counts of MIWI2 piRNAs
sense piRNAs KI/KI
antisense piRNAs KI/KI
−/−
+/KI
LINE
LTR
SINE
D
SINE
LTR
LINE
MILI piRNAs
MIWI2 piRNAs
sense
antisense
piRNAs (rpm)
1U & 10A
only 1U
only 10A
other
+/KI
KI/KI
−/−

Figure- S8

Supplemental Table S1. List of all deep-sequencing libraries created in this study. Related to STAR Methods and Figure 1.

Sample	Description	Mapped to	Mapped reads
RR390	Rosa26-pi reporter;MVH +/-;P0;MILI IP;piRNAs	DsRed2 reporter	29048
RR392	Rosa26-pi reporter;MVH +/KI;P0;MILI IP;piRNAs	DsRed2 reporter	41403
RR393	Rosa26-pi reporter;MVH -/KI;P0;MILI IP;piRNAs	DsRed2 reporter	6044
RR391	Rosa26-pi reporter;MVH -/-;P0;MILI IP;piRNAs	DsRed2 reporter	13799
RR394	Rosa26-pi reporter;MVH +/-;P0;MILI IP;longer RNAs	DsRed2 reporter	7472
RR396	Rosa26-pi reporter;MVH +/KI;P0;MILI IP;longer RNAs	DsRed2 reporter	8800
RR397	Rosa26-pi reporter;MVH -/KI;P0;MILI IP;longer RNAs	DsRed2 reporter	1030
RR395	Rosa26-pi reporter;MVH -/-;P0;MILI IP;longer RNAs	DsRed2 reporter	3580
RR374	MVH +/-;P0;MILI IP;piRNAs	mm9 genome	15626151
RR375	MVH +/KI;P0;MILI IP;piRNAs	mm9 genome	13538148
RR376	MVH -/KI;P0;MILI IP;piRNAs	mm9 genome	12703627
RR377	MVH +/-;P0;MIWI2 IP;piRNAs	mm9 genome	14576415
RR378	MVH +/KI;P0;MIWI2 IP;piRNAs	mm9 genome	10718339
RR379	MVH +/-;P0;MILI IP;longer RNAs	mm9 genome	15514252
RR380	MVH +/KI;P0;MILI IP;longer RNAs	mm9 genome	8363415
RR381	MVH -/KI;P0;MILI IP;longer RNAs	mm9 genome	10160004
RR382	MVH +/-;P0;MIWI2 IP;longer RNAs	mm9 genome	12118125
RR383	MVH +/KI;P0;MIWI2 IP;longer RNAs	mm9 genome	8179838
JW9	MVH +/KI;adult;MIWI IP;piRNAs	mm9 genome	29122251
JW10	MVH +/-;adult;MIWI IP;piRNAs	mm9 genome	31886830
JW11	MVH +/KI;adult;MILI IP;piRNAs	mm9 genome	23647521
JW12	MVH +/-;adult;MILI IP;piRNAs	mm9 genome	32987116
JW13	MVH +/KI;adult;MVH IP;piRNAs;rep1	mm9 genome	24350164
JW14	MVH +/KI;adult;MVH IP;piRNAs;rep2	mm9 genome	23031757
JW15	MVH +/KI;adult;MVH IP;piRNAs;rep3	mm9 genome	34031418
JW16	MVH +/+;adult;MVH IP;piRNAs	mm9 genome	10861787
JW17	MVH +/KI;adult;MIWI IP;longer RNAs	mm9 genome	28616395
JW18	MVH +/-;adult;MIWI IP;longer RNAs	mm9 genome	22442466
JW19	MVH +/KI;adult;MILI IP;longer RNAs	mm9 genome	13072246
JW20	MVH +/-;adult;MILI IP;longer RNAs	mm9 genome	22496088
JW21	MVH +/KI;adult;MVH IP;longer RNAs;rep1	mm9 genome	20016795
JW22	MVH +/KI;adult;MVH IP;longer RNAs;rep2	mm9 genome	18634953
JW23	MVH +/KI;adult;MVH IP;longer RNAs;rep3	mm9 genome	51730274
JW24	MVH +/+;adult;MVH IP;longer RNAs	mm9 genome	20429840
RR301	Tdrd9 +/KI;P0;MILI IP;piRNAs	mm9 genome	10371936
RR302	Tdrd9 KI/KI;P0;MILI IP;piRNAs	mm9 genome	11651344
RR303	Tdrd9 -/-;P0;MILI IP;piRNAs	mm9 genome	12228347
RR304	Tdrd9 +/KI;P0;MIWI2 IP;piRNAs	mm9 genome	16424774
RR305	Tdrd9 KI/KI;P0;MIWI2 IP;piRNAs	mm9 genome	14003398
RR306	Tdrd9 -/-;P0;MIWI2 IP;piRNAs	mm9 genome	11133876

Supplemental Table 2. List of the top 50 mouse pachytene piRNA clusters used for data analysis. Related to STAR Methods and Figure 4.

#	chr	chr_start	chr_end
1	chr17	27427000	27505001
2	chr9	67538000	67615001
3	chr2	92374000	92452001
4	chr9	54047000	54112001
5	chr6	127726000	127801001
6	chr12	99618000	99694001
7	chr18	67188000	67259001
8	chr15	59078000	59136001
9	chr7	80915000	80974001
10	chr14	24898000	24946001
11	chr5	150591000	150636001
12	chr7	77021000	77101001
13	chr5	113745000	113793001
14	chr17	66536000	66587001
15	chr7	80241000	80298001
16	chr15	74459000	74497001
17	chr4	93942000	94000001
18	chr10	75298000	75326001
19	chr8	112641000	112656001
20	chr6	81843000	81854001
21	chr10	62114000	62158001
22	chr11	103284000	103313001
23	chr8	94712000	94724001
24	chr5	115283000	115306001
25	chr14	20444000	20473001
26	chr15	78468000	78502001
27	chr4	57368000	57379001
28	chr7	132467000	132479001
29	chr1	94887000	94895001
30	chr7	60161000	60174001
31	chr10	85194000	85232001
32	chr15	83183000	83197001
33	chr10	66144000	66173001
34	chr4	61880000	61894001
35	chr3	124102000	124126001
36	chr6	128131000	128143001
37	chr6	85060000	85067001
38	chr8	95933000	95949001
39	chr10	18515000	18527001
40	chr4	135181000	135187001
41	chr3	34765000	34779001
42	chr6	87926000	87945001
43	chr13	53488000	53500001
44	chr1	93450000	93457001
45	chr14	25228000	25250001
46	chr10	86027000	86058001
47	chr11	107854000	107861001
48	chr5	144523000	144535001
49	chr17	66517000	66533001
50	chr7	30917000	30926001

the positions refer to mm9 coordinates
